# Mitofusin-2 in ventral striatal D1 neurons regulates effort-based motivation through sex-specific mitochondrial–synaptic reprogramming

**DOI:** 10.1073/pnas.2601657123

**Published:** 2026-07-15

**Authors:** Alessandro Chioino, Dogukan H. Ulgen, Olivia Zanoletti, Isabelle Guillot de Suduiraut, Ashley M. Maynard, Elisenda Sanz, Albert Quintana, Simone Astori, Carmen Sandi

**Affiliations:** ^a^ Laboratory of Behavioral Genetics, Brain Mind Institute, School of Life Sciences, Ecole Polytechnique Fédérale de Lausanne, Lausanne 1015, Switzerland; ^b^ Synapsy Center for Neuroscience and Mental Health Research, School of Life Sciences, Ecole Polytechnique Fédérale de Lausanne, Lausanne 1015, Switzerland; ^c^ Regeneration and Neurogenomics Laboratory, Brain Mind Institute, School of Life Sciences, Ecole Polytechnique Fédérale de Lausanne, Lausanne 1015, Switzerland; ^d^ Institut de Neurociències, Universitat Autònoma de Barcelona, Bellaterra 08193, Spain; ^e^ Departament de Biologia Cellular, Fisiologia i Immunologia, Universitat Autònoma de Barcelona, Barcelona 08193, Spain; ^f^ Focus Area for Human Metabolomics, Faculty of Natural and Agricultural Sciences, North-West University, Potchefstroom 2520, South Africa

**Keywords:** mitochondria, motivation, ventral striatum

## Abstract

Effort-based motivation varies widely across individuals and affects well-being, yet the molecular and neuronal mechanisms that set motivational capacity remain incompletely understood. Mitochondrial function is emerging as a critical regulator of behavior, and mitofusin-2 (MFN2) is a key mediator of mitochondrial fusion and endoplasmic reticulum–mitochondria coupling. Here, we asked how downregulation of *Mfn2* in dopamine receptor type-1-expressing medium spiny neurons (D1-MSNs) contributes to effort-based motivation and stress coping in male and female mice by integrating electrophysiology, neuronal and synaptic morphology, immunohistochemistry, mitochondrial readouts, RNA in situ hybridization, RiboTag, and behavioral analyses. MFN2 deficiency resulted in fragmented dendritic mitochondria and remodeled synaptic inputs in ventral striatal D1-MSNs, with no cellular impact in dorsomedial striatal D1-MSNs. Although MFN2 deficiency elicited sex-dependent synaptic and structural alterations, both sexes showed reduced recruitment of accumbal D1-MSNs during motivated behavior and impaired effort-based motivation and stress coping. Translatome profiling revealed shared depletion of mitochondrial pathways in both sexes, with more pronounced suppression of oxidative phosphorylation and TCA cycle programs in males. Strikingly, only males also exhibited coordinated downregulation of ribosomal programs together with enrichment of synaptic pathways, with high representation of genes regulating glutamate receptor cycling and PSD remodeling, providing a mechanistic framework for the observed synaptic alterations. Pathway-level network inference further supported coupling among mitochondrial, translational, and synaptic programs in males. These findings identify MFN2-dependent mitochondrial integrity in ventral striatal D1-MSNs as a critical determinant of motivational capacity and reveal sex-specific molecular and cellular responses through which mitochondrial dysfunction converges on similar motivational deficits.

## Significance

Effort-based motivation, the willingness to invest work to achieve rewards, varies widely in the population and, when blunted, contributes to depression, apathy, and loss of productivity. We identify mechanisms driven by alterations in mitochondrial integrity in ventral striatum dopamine receptor 1 (D1)-expressing neurons, a core reward-circuit cell type, that constrain this capacity. A deficit in the expression of the mitochondrial fusion protein mitofusin-2 fragments mitochondria, reshapes synaptic inputs, and reduces recruitment of these neurons during motivated behavior, impairing effortful behavior and active stress coping. Males and females reach similar behavioral deficits through distinct molecular programs, revealing sex-specific routes from mitochondrial dysfunction to motivational impairment and supporting sex-informed and circuit-targeted mitochondrial interventions.

Motivation is a fundamental behavioral process enabling individuals to overcome effort-related costs to achieve desired outcomes, critically influenced by specific cellular and molecular mechanisms (1–3) and impacting well-being, productivity, and adaptive functioning (4–6). Substantial individual differences exist in motivational capacity, manifesting as distinct patterns of effort allocation and reward-seeking behavior (7–9). Dysfunction in motivational processes is a hallmark feature of several psychiatric and neurological conditions, including depression and apathy (10, 11), highlighting the importance of elucidating their underlying neurobiological mechanisms.

Central to the neural circuits regulating motivation is the nucleus accumbens (NAc), a part of the ventral striatum whose medium spiny neurons (MSNs) integrate multiple synaptic inputs to orchestrate behavioral responses essential for reward-seeking and effort exertion (1, 12–14). Structural and functional alterations within the NAc have been consistently linked to deficits in reward processing and motivation (1, 15–21) in both animals and humans. Within the NAc, MSNs are classified into two main subtypes based on their relative expression of dopamine receptor D1 or D2. D1-MSNs have been classically implicated in promoting motivated behaviors and encoding reward value and effort costs (22–25), while D2-MSNs are more often related to aversion and inhibitory processes (26–28). Disruptions in their respective engagement during behavioral states have been implicated in vulnerability to stress and depression (19). Although recent evidence indicates that both D1- and D2-MSNs can cooperatively encode motivational states, with their specific contributions to reward or aversion possibly determined by their distinct firing patterns (29–31), significant gaps remain in understanding the molecular and cellular mechanisms within these neuronal populations that specifically influence effort-based motivational functions.

Emerging evidence highlights the role of mitochondrial function and metabolism within the NAc in shaping motivated behaviors (32–37). Mitochondria dynamically regulate neuronal energy metabolism, Ca^2+^ buffering, and synaptic plasticity (38–40), processes essential to sustaining neuronal and circuit-level functions underlying motivated behavior. Mitofusin-2 (MFN2) is a key mitochondrial outer membrane GTPase mediating mitochondrial fusion (38, 41), and maintaining functional coupling between mitochondria and the endoplasmic reticulum (ER), thereby influencing mitochondrial morphology, metabolic integrity, and Ca^2+^ homeostasis (38, 42–45). Our prior work showed that natural variation in accumbal *Mfn2* expression is linked to differences in motivational performance and anxiety-like behaviors in rodents and that restoring *Mfn2* levels in accumbal MSNs of naturally high-anxious animals with lower *Mfn2* levels, is sufficient to normalize the associated mitochondrial, neuronal, and behavioral alterations (35, 46). Moreover, selective MFN2 deficiency in NAc D2-MSNs impairs mitochondrial bioenergetics, neuronal excitability, and social dominance behaviors (46).

However, key gaps remain in understanding the specific role of *Mfn2* in D1-MSNs. First, it is unknown whether and how *Mfn2* modulates reward- and effort-based motivated behaviors, extending beyond its previously reported role in aversive motivation (35). Second, most work to date has focused on males, leaving potential sex-dependent influences of *Mfn2* unresolved. Third, the gene-expression programs altered by MFN2 deficiency in D1-MSNs, and their relationship to changes in neuronal function, remain largely uncharacterized, limiting our understanding of the underlying molecular pathways and hindering the development of targeted interventions.

Addressing these gaps, we selectively downregulated *Mfn2* in NAc D1-MSNs in male and female mice and integrated mitochondrial, electrophysiological, morphological, transcriptomic, and behavioral analyses to define how *Mfn2* loss affects neuronal function and effort-based motivation. This approach revealed both shared and sex-specific adaptations, providing key insights into mitochondrial contributions to motivational regulation within defined neuronal populations.

## Results

### The Impact of *Mfn2* Deficiency in D1-MSNs Is Region Specific.

**Fig. 1.**
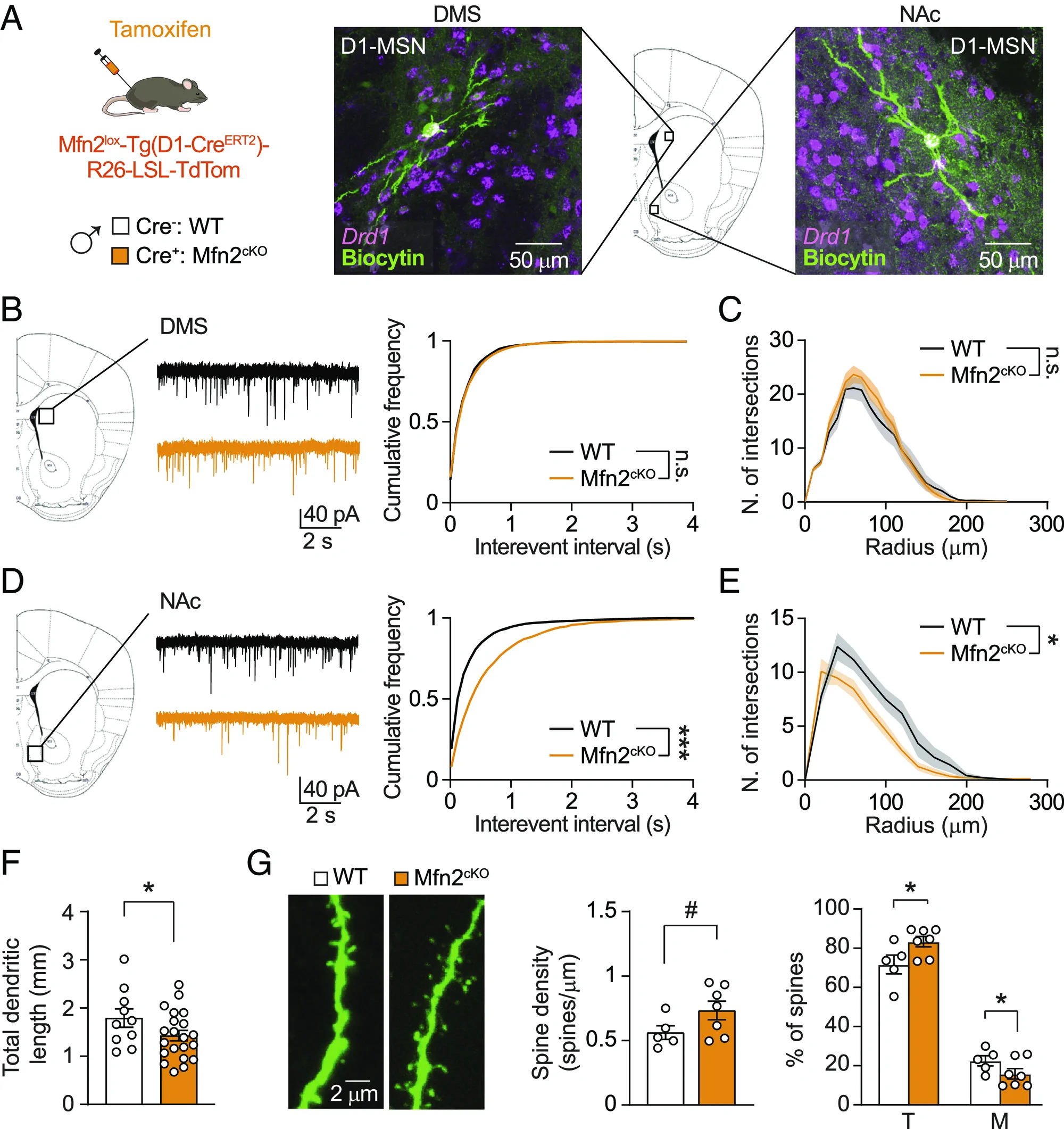
Regional specificity of *Mfn2* deficiency effects within striatal D1-MSN populations. (*A*) Experimental design for tamoxifen-induced Cre^ERT2^-mediated conditional *Mfn2* deletion specifically in D1-expressing cells (Mfn2^cKO^), with example images of biocytin-filled D1-MSNs (*Drd*1-positive) in the dorsomedial striatum (DMS) and the NAc. (*B*) Representative traces of mEPSC recordings in DMS D1-MSNs (*Left*), and cumulative frequency of interevent intervals (*Right*), indicating no change in excitatory input frequency in Mfn2^cKO^ mice (Kolmogorov–Smirnov D = 0.029, *P* = 0.33; n = 10 cells in 4 WT; n = 12 cells in 5 Mfn2^cKO^). (*C*) Sholl analysis in DMS D1-MSNs, showing unaltered dendritic complexity in Mfn2^cKO^ mice (Mixed effect model for effect genotype F_(1, 15)_ = 0.35, *P* = 0.56; n = 8 cells in 3 WT; n = 9 cells in 4 Mfn2^cKO^). (*D*) Representative traces of mEPSC recordings in NAc D1-MSNs (*Left*), and cumulative frequency of interevent intervals (*Right*), indicating reduced excitatory input frequency in Mfn2^KO^ mice (Kolmogorov–Smirnov D = 0.29, *P* < 0.0001; n = 7 cells in 3 WT; n = 10 cells in 5 Mfn2^cKO^). (*E*) Sholl analysis in NAc D1-MSNs, showing reduced dendritic complexity in Mfn2^cKO^ mice (Mixed effect model for effect genotype F_(1, 29)_ = 4.8, *P* = 0.04; n = 8 cells in 3 WT; n = 9 cells in 4 Mfn2^cKO^). (*F*) Total dendritic length showing significant reduction in NAc D1-MSNs from Mfn2^cKO^ mice (unpaired *t* test, *P* = 0.04; n = 10 cells in 5 WT; n = 21 cells in 12 Mfn2^cKO^). (*G*) Spine morphology analysis indicating higher spine density (*Left*, unpaired *t* test, *P* = 0.054), with an increased proportion of thin spines and reduced mushroom spines (*Right*, Mann–Whitney test, *P* = 0.023; n = 5 cells in 3 WT; n = 7 cells in 5 Mfn2^cKO^) in NAc D1-MSNs from Mfn2^cKO^ mice.

To investigate the cellular impact of MFN2 in D1-MSNs, we first assessed whether its function is region-specific by examining the effects of *Mfn2* deficiency in two major striatal regions, the NAc and the dorsomedial striatum (DMS). To this end, we used a tamoxifen-induced Cre^ERT2^ strategy allowing for conditional deletion of *Mfn2* in D1-expressing neurons (hereafter referred to as Mfn2^cKO^), and assessed the electrophysiological responses in ex vivo patch-clamp recordings from D1-MSNs in the NAc and DMS from male mice (Fig. 1*A*). This approach allows for homogeneous downregulation of *Mfn2* across all D1-expressing neurons, providing an optimal strategy for assessing cell-autonomous effects of Mfn2 loss.

First, we recorded miniature excitatory postsynaptic currents (mEPSCs) in the presence of the Na_v_ channel blocker tetrodotoxin and the GABA_A_R blocker picrotoxin, and performed biocytin filling to allow for morphological reconstructions. Mfn2^cKO^ in D1-MSNs from the DMS did not affect mEPSCs frequency or dendritic complexity (Fig. 1 *B* and *C*). By contrast, NAc D1-MSNs from Mfn2^cKO^ mice displayed a prolonged interevent interval of mEPSCs (Fig. 1*D*). In addition, Mfn2^cKO^ reduced the dendritic complexity in NAc D1-MSNs (Fig. 1 *E* and *F*), and increased the proportion of thin spines compared to mushroom spines, indicating a shift toward less mature synaptic connections (Fig. 1*G*).

Collectively, these findings indicate a region-specific impact of *Mfn2* deficiency, manifested as alterations in synaptic input dynamics, dendritic and spine morphology that, within the striatum, appear specific to NAc D1-MSNs. Building on this cell-type and region-specific vulnerability, we next sought to determine whether *Mfn2* deficiency in NAc D1-MSNs translates into functional behavioral consequences in vivo.

### AAV-mediated Mfn2KO Engages Core Mfn2-dependent Mitochondrial/ER Functions in D1-MSNs.

After establishing that *Mfn2* deficiency selectively affects D1-MSNs in the NAc, but not in the DMS, with the tamoxifen-inducible Mfn2^cKO^ approach, we moved to an AAV-mediated regional knockout strategy to test the behavioral consequences of *Mfn2* loss specifically in accumbal D1-MSNs. To delete *Mfn2* specifically in NAc D1-MSNs, we employed an AAV-mediated knockout approach that selectively and efficiently targeted these neurons in Mfn2^lox/lox^ mice (*SI Appendix*, Fig. S1), hereafter referred to as Mfn2^KO^. We next used complementary ultrastructural and functional readouts to verify that Mfn2^KO^ engages core MFN2-dependent mitochondrial and mitochondria–ER functions (38–40). 3D reconstructions of NAc D1-MSN dendritic mitochondria using confocal and correlated light and electron microscopy (CLEM), showed smaller, more spherical, and less complex mitochondria, together with reduced mitochondria–ER contacts (*SI Appendix*, Fig. S2 *A*–*H*). In primary striatal cultures, Mfn2^KO^ also impaired mitochondrial Ca^2+^ uptake, as shown by reduced mitoGCaMP responses to KCl-induced depolarization, whereas mitochondrial membrane potential assessed with TMRM was largely unchanged (*SI Appendix*, Fig. S2 *I* and *J*). Thus, AAV-mediated Mfn2^KO^ disrupts mitochondrial morphology, ER-contact organization, and mitochondrial Ca^2+^ handling, without evidence for generalized loss of mitochondrial membrane potential. Because the CLEM analyses were performed in males and the primary culture assays involved mixed-sex neonatal cultures and, thus, were not sex-stratified, these data are used as validation of the Mfn2^KO^ manipulation rather than as a sex-comparative readout.

### *Mfn2* Deficiency in Accumbal D1-MSNs Impairs Motivated Behavior and Associated Neuronal Activation in Male Mice.

**Fig. 2.**
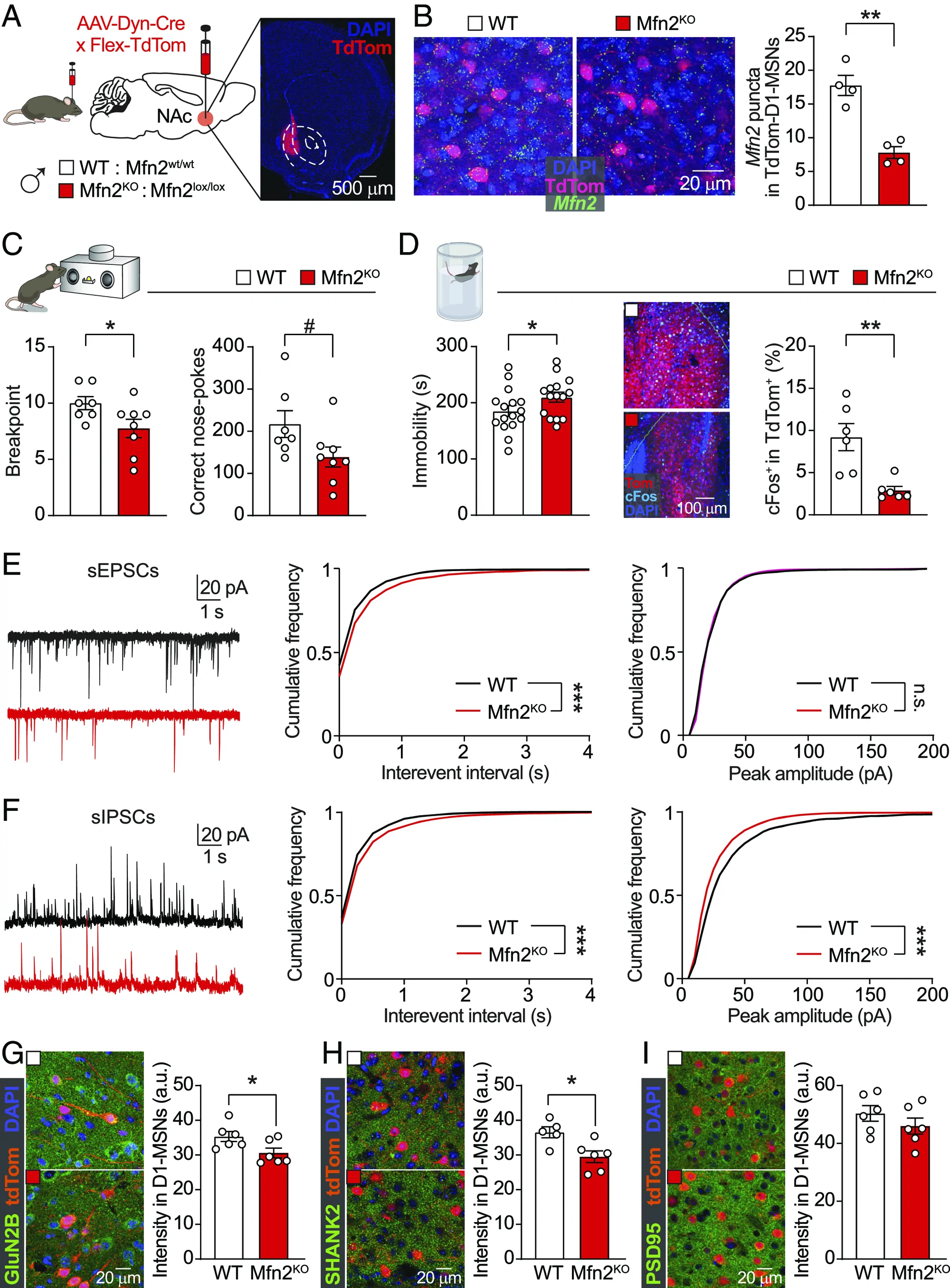
*Mfn2* deficiency in D1-MSNs impairs motivated behaviors and associated neuronal activation. (*A*) Experimental schematic depicting viral-mediated Mfn2^KO^ selectively in D1-MSNs in the NAc. (*B*) Representative RNAscope images and quantification showing reduced *Mfn2* puncta in TdTomato-labeled D1-MSNs in Mfn2^KO^ mice (unpaired *t* test, *P* = 0.001). (*C*) Performance in the progressive ratio (PR) task, illustrating significantly lower breakpoint (*Left*, unpaired *t* test, *P* = 0.04) and a trend toward fewer correct nose-pokes (*Right*, unpaired *t* test, *P* = 0.07) in Mfn2^KO^ mice. (*D*) Forced swim test (FST) showing significantly increased immobility in Mfn2^KO^ mice (unpaired *t* test, *P* = 0.04) and representative images with quantification of cFos immunoreactivity within D1-MSNs, indicating significantly lower neuronal activation posttask in Mfn2^KO^ mice (Mann–Whitney test, *P* = 0.009). (*E*) Representative traces of sEPSC recordings (*Left*), and cumulative frequency of interevent intervals (*Center*), and peak amplitude (*Right*), highlighting reduced excitatory input frequency (Kolmogorov–Smirnov D = 0.09788, *P* < 0.0001) but unchanged amplitude in Mfn2^KO^ neurons (Kolmogorov–Smirnov D = 0.05, *P* > 0.001; n = 10 cells in 6 WT; n = 13 cells in 5 Mfn2^KO^). (*F*) Representative traces of sIPSC recordings (*Left*), and cumulative frequency of interevent intervals (*Center*), and peak amplitude (*Right*), showing reduced inhibitory input frequency (Kolmogorov–Smirnov D = 0.084, *P* < 0.0001) and decreased amplitude in Mfn2^KO^ neurons (Kolmogorov–Smirnov D = 0.14, *P* < 0.0001; n = 10 cells in 6 WT; n = 13 cells in 5 Mfn2^KO^). (*G*–*I*) Representative immunohistochemical images and quantification of GluN2B (unpaired *t* test, *P* = 0.039), Shank2 (unpaired *t* test, *P* = 0.015), and PSD95 (unpaired *t* test, *P* = 0.286) in TdTomato-labeled D1-MSNs, indicating decrease of synaptic proteins in Mfn2^KO^ neurons.

Having established that the AAV-mediated Mfn2^KO^ strategy enables selective targeting of NAc D1-MSNs and engages core *Mfn2*-dependent mitochondrial and mitochondria–ER functions, we next tested whether this manipulation affects motivated behavior in male mice. We first confirmed successful targeting of the viral injection (Fig. 2*A*) and reduction of *Mfn2* expression in the targeted population using RNAscope, observing fewer *Mfn2* puncta in TdTomato-labeled NAc D1-MSNs in Mfn2^KO^ mice compared with WT controls (Fig. 2*B*).

To test whether selective *Mfn2* deficiency in accumbal D1-MSNs impacts motivated behaviors, we subjected WT and Mfn2^KO^ male mice to effort-based motivation tasks (*SI Appendix*, Fig. S3*A*, experimental scheme) to investigate reward- and effort-based motivational capacity, alongside the FST, which assesses stress coping responses that engage motivational processes under aversive conditions. Behaviorally, Mfn2^KO^ mice displayed significantly reduced motivation as indicated by a lower breakpoint in the PR task compared to controls (Fig. 2 *C*, *Left*). There was also a trend toward fewer correct nose-pokes in Mfn2^KO^ mice, indicating diminished reward-directed responses (Fig. 2 *C*, *Right*). These deficits were not due to general performance or learning impairments, as Mfn2^KO^ mice showed normal free feeding behavior and comparable reward acquisition in fixed-ratio 1 (FR1) schedules (*SI Appendix*, Fig. S3 *B* and *C*), indicating that Mfn2^KO^ did not affect basic food motivation, motor abilities, or task acquisition. In contrast, performance deficits emerged during more demanding FR3 sessions (*SI Appendix*, Fig. S3*D*) and persisted over repeated PR tasks (*SI Appendix*, Fig. S3*E*), further confirming reduced motivational drive in Mfn2^KO^ animals.

Additionally, we observed significantly higher immobility in Mfn2^KO^ mice during the FST, consistent with increased passive coping behavior and reduced engagement of motivational processes under acute stress (Fig. 2 *D*, *Left*). To link these behavioral impairments with neuronal activation, we assessed posttask cFos expression specifically within D1-MSNs in the NAc, which was significantly reduced in Mfn2^KO^ mice, indicating reduced recruitment of mitochondrially impaired D1-MSNs during motivated behavior (Fig. 2 *D*, *Right*). Additional behavioral analyses indicated that these motivational alterations were not explained by impaired locomotor activity, increased anxiety-like behavior, or altered novelty exploration (*SI Appendix*, Fig. S4).

To investigate how *Mfn2* deficiency in accumbal D1-MSNs impacts synaptic integration and neuronal structure, we performed ex vivo patch-clamp recordings and morphological reconstructions in the NAc shell. Using a sequential voltage-clamp protocol, we recorded spontaneous excitatory (sEPSCs) and inhibitory postsynaptic currents (sIPSCs) from the same neurons (Fig. 2 *E* and *F*). Mfn2^KO^ neurons showed reduced excitatory input frequency, indicated by a rightward shift in the cumulative distribution of sEPSC interevent interval. These results are consistent with the reduced mEPSC frequency recorded in Mfn2^cKO^ mice (Fig. 1*D*). The more pronounced electrophysiological phenotype observed in Mfn2^cKO^ mice likely arises from a combination of methodological factors, including the isolation of quantal, action potential-independent events in the presence of tetrodotoxin and the more homogeneous *Mfn2* deletion achieved with the tamoxifen-inducible conditional knockout relative to the virally mediated approach. The amplitude distribution of sEPSCs was unchanged, suggesting preserved postsynaptic responsiveness (Fig. 2*E*). Conversely, sIPSCs occurred less frequently and displayed lower peak amplitudes in Mfn2^KO^ neurons (Fig. 2*F*), consistent with reduced inhibitory synaptic input. To investigate whether these synaptic alterations in mitochondrial dynamics were associated with changes in synaptic organization, we next assessed the expression of key excitatory postsynaptic proteins in TdTomato-labeled D1-MSNs. Immunohistochemical analyses revealed a significant reduction in GluN2B and SHANK2 levels in Mfn2^KO^ neurons compared to WT controls, whereas PSD95 expression was not significantly altered (Fig. 2 *G*–*I*). These findings indicate that *Mfn2* deficiency selectively disrupts components of the postsynaptic excitatory machinery in accumbal D1-MSNs, consistent with impaired synaptic integrity and reduced excitatory connectivity.

Collectively, these behavioral, neuronal activation, electrophysiological, and protein-level findings indicate that selective *Mfn2* deficiency in male accumbal D1-MSNs impairs effort-based motivation and active stress coping, reduces recruitment of the targeted neuronal population during motivated behavior, and disrupts synaptic input dynamics and postsynaptic organization.

### Female Mice Exhibit Similar Motivational Deficits but Distinct Synaptic and Morphological Alterations Upon Accumbal *Mfn2* Deficiency.

**Fig. 3.**
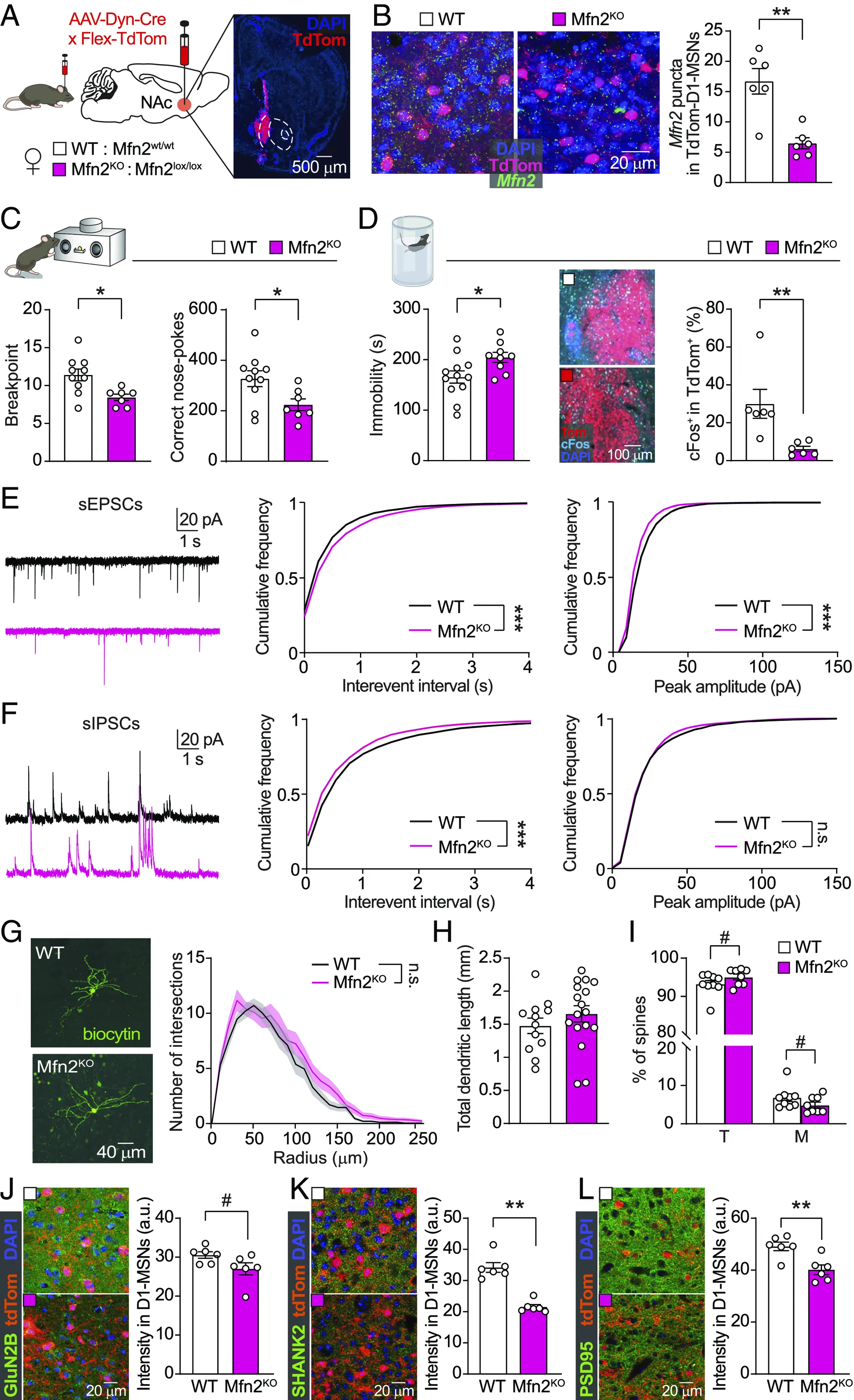
Motivational deficits and synaptic and morphological alterations in female mice upon Mfn2^KO^. (*A* and *B*) Experimental design and RNAscope confirmation of reduced Mfn2 puncta in TdTomato-labeled D1-MSNs from female Mfn2^KO^ mice (unpaired *t* test, *P* = 0.001). (*C*) PR task showing significantly lower breakpoint (unpaired *t* test, *P* = 0.005) and fewer nose-pokes (unpaired *t* test, *P* = 0.019) in female Mfn2^KO^ mice. (*D*) FST demonstrating increased immobility in female Mfn2^KO^ mice compared to WT controls (*Left*, unpaired *t* test, *P* = 0.023). Quantification of cFos immunoreactivity within D1-MSNs, indicating significantly lower neuronal activation posttask in Mfn2^KO^ female mice (*Right*, Mann–Whitney test, *P* = 0.002). (*E*) Representative traces of sEPSC recordings (*Left*), and cumulative frequency of interevent intervals (*Center*), and peak amplitude (*Right*), highlighting reduced excitatory input frequency (Kolmogorov–Smirnov D = 0.077, *P* < 0.0001) and decreased amplitude in Mfn2^KO^ neurons (Kolmogorov–Smirnov D = 0.14, *P* < 0.0001; n = 11 cells in 3 WT; n = 16 cells in 4 Mfn2^KO^). (*F*) Representative traces of sIPSC recordings (*Left*), and cumulative frequency of interevent intervals (*Center*), and peak amplitude (*Right*), showing reduced inhibitory input frequency (Kolmogorov–Smirnov D = 0.093, *P* < 0.0001) with unaltered amplitude in Mfn2^KO^ neurons (Kolmogorov–Smirnov D = 0.036, *P* = 0.07; n = 11 cells in 3 WT; n = 16 cells in 4 Mfn2^KO^). (*G*) Sholl analysis in NAc D1-MSNs, showing unaltered dendritic complexity in Mfn2^KO^ female mice (Mixed effect model for effect genotype F_(1, 26)_ = 1.5, *P* = 0.23; n = 12 cells in 4 WT; n = 16 cells in 4 Mfn2^KO^). (*H*) Total dendritic length showing no change in NAc D1-MSNs from Mfn2^KO^ female mice (unpaired *t* test, *P* = 0.157; n = 12 cells in 4 WT; n = 17 cells in 4 Mfn2^KO^). (*I*) Spine morphology analysis indicating a tendency to increased proportion of thin spines and reduced mushroom spines in NAc D1-MSNs from Mfn2^KO^ mice (Mann–Whitney test, *P* = 0.098; n = 9 cells in 4 WT; n = 8 cells in 4 Mfn2^KO^). (*J*–*L*) Representative immunohistochemical images and quantification of GluN2B (unpaired *t* test, *P* = 0.072), Shank2 (Mann–Whitney test, *P* = 0.002), and PSD95 (unpaired *t* test, *P* = 0.005) in TdTomato-labeled D1-MSNs, indicating decrease of synaptic proteins in Mfn2^KO^ neurons.

To determine whether the behavioral and cellular consequences of accumbal *Mfn2* deficiency observed in males are conserved in females, we conducted parallel experiments in female mice using the same AAV-mediated regional knockout strategy (Fig. 3*A*). Similar to males, female Mfn2^KO^ mice exhibited robust reductions in *Mfn2* puncta in TdTomato-labeled D1-MSNs (Fig. 3*B*). Behaviorally, female Mfn2^KO^ mice displayed impaired motivated behaviors, evidenced by significantly lower breakpoints during the PR task compared to WT controls (Fig. 3*C*). Complementary analyses showed normal free-feeding pellet consumption and no significant differences in simpler reward tasks such as FR1 and FR3 schedules (*SI Appendix*, Fig. S5 *A*–*D*). PR performance on day 2 also confirmed a reduced breakpoint in Mfn2^KO^ females (*SI Appendix*, Fig. S5*E*). In the FST, female Mfn2^KO^ mice showed significantly increased immobility compared to WT, indicating reduced motivational resilience similar to males, and this was accompanied by reduced cFos activation in accumbal D1-MSNs (Fig. 3*D*). Additional behavioral analyses indicated no difference in locomotor activity and anxiety levels between groups (*SI Appendix*, Fig. S6) and, therefore, their motivational and coping phenotypes were not explained by locomotor, anxiety-like, novelty exploration or task acquisition confounds.

Electrophysiological recordings from NAc shell D1-MSNs revealed that Mfn2^KO^ females exhibited distinct synaptic changes compared to males. Female Mfn2^KO^ neurons showed reduced sEPSC frequency (increased interevent intervals) and lower sEPSC amplitudes (Fig. 3*E*). In contrast, inhibitory inputs were more frequent, while sIPSC amplitudes remained unchanged (Fig. 3*F*). Morphologically, female Mfn2^KO^ neurons showed no detectable changes in dendritic arborization (Fig. 3*G*) or total dendritic length (Fig. 3*H*). However, the profile of synaptic spines indicated a shift toward a higher proportion of immature spines (Fig. 3*I*), similar to the findings from male mice. In addition, as in male Mfn2^KO^, immunohistochemical analyses revealed reductions in postsynaptic excitatory components, including significant decreases in SHANK2 and PSD95 and a trend toward reduced GluN2B (Fig. 3 *J*–*L*), consistent with impaired synaptic organization and reduced excitatory synaptic integrity.

Together, these findings indicate that, while motivational impairments due to *Mfn2* deficiency in D1-MSNs are similar across sexes, the underlying synaptic and structural adaptations show sex-specific patterns.

### *Mfn2* Deficiency Reprograms Mitochondrial and Synaptic Translatomes in NAc D1-MSNs in a Sex-Specific Manner.

To gain insight into the molecular changes induced by accumbal *Mfn2* deficiency (Mfn2^KO^), we carried out RiboTag RNA sequencing on D1-MSNs expressing the HA-tagged ribosomal subunit RPL22, thereby obtaining the ribosome-bound mRNAs in a cell-specific manner (47, 48) in both males and females. The cell-type specificity of the RiboTag approach (*SI Appendix*, Fig. S7*A*) and the efficiency of the Mfn2^KO^ (*SI Appendix*, Fig. S7 *B*–*E*) were confirmed. There were 34 differentially expressed genes (DEGs) in the Mfn2^KO^ vs. WT comparisons in each sex: 17 upregulated and 17 downregulated transcripts in males (*SI Appendix*, Fig. S7*F*) and 19 upregulated and 15 downregulated transcripts in females (*SI Appendix*, Fig. S7*G* and Table S1 in Dataset S1), while experimental groups did not separate distinctly in PCA space (*SI Appendix*, Fig. S7 *H* and *I*).

**Fig. 4.**
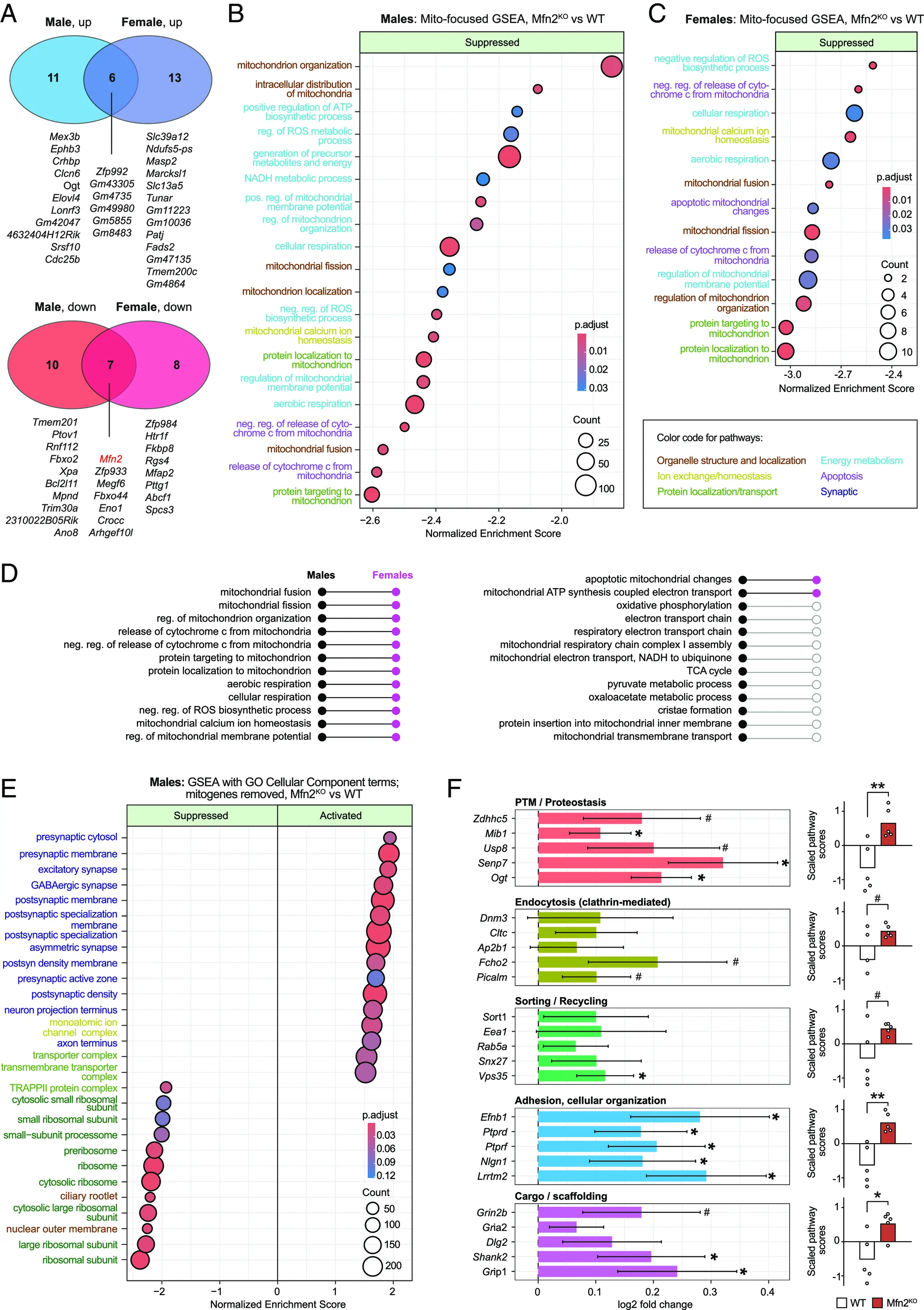
RiboTag analysis reveals translatome changes in accumbal D1-MSNs upon Mfn2^KO^. (*A*) Venn diagrams showing shared and nonshared DEGs in males and females; for upregulated (*Above*) and downregulated (*Below*) genes, with the gene names indicated below (males: n = 10 with 5 WT and 5 Mfn2^KO^; females: n = 10 with 5 WT and 5 Mfn2^KO^). (*B*) Male mito-focused GSEA results. Mitochondria-related GO-BP terms were chosen based on overlap with genes with mitochondrial function (>50%) and gene-set size (>10). Pathways are color-coded by relevance as organelle structural and localization changes (brown), energy metabolism (cyan), ion exchange/homeostasis (light green), apoptosis (purple), and protein localization (green). (*C*) Female mito-focused GSEA results. Pathways are color-coded by relevance as in panel *B*. (*D*) Comparison of mito-focused GSEA results between males and females. Filled circles indicate significant enrichments for the indicated sex. (*E*) Male GSEA results after genes with mitochondrial function were removed. Pathways are color-coded by relevance as synaptic (dark blue), ion exchange/homeostasis (light green), protein localization (green), and ribosomal (dark green). (*F*) (*Left*) Bar plots for genes of curated synaptic functions. Normalized gene counts are scaled. (*Right*) Composite scores for the curated pathways, calculated as the mean of scaled normalized gene counts. *P < 0.05, **P < 0.01, ^#^*P* < 0.1.

Comparing the general changes induced by Mfn2^KO^ in males and females, we identified 13 DEGs shared between sexes (6 upregulated, 7 downregulated), while two nonoverlapping sets of 21 DEGs each were specific to males or females (Fig. 4*A*). Shared genes include components of ciliary and centrosomal architecture (*Crocc*), glycolytic metabolism (*Eno1*), ubiquitin-ligase substrate recognition (*Fbxo44*), extracellular signaling and matrix-associated factors (*Megf6*), and KRAB zinc-finger proteins (*Zfp992*, *Zfp933*), suggesting adaptations in structural organization, metabolic capacity, and gene-regulatory programs (49, 50). Male-specific DEGs included genes involved in synaptic connectivity and glycoprotein turnover (*Fbxo2*), neuronal endolysosomal function (*Clcn6*), proapoptotic signaling (*Bcl2l11*), DNA repair (*Xpa*), nutrient-sensitive O-GlcNAc signaling (*Ogt*), and very long-chain fatty acid synthesis for neuronal membranes (*Elovl4*), suggestive of a stress- and damage-related transcriptional response (51–54). In contrast, female-specific DEGs included genes related to lipid metabolism and citrate transport (*Fads2*, *Slc13a5*), synaptic and tight junction scaffolding and signaling (*Patj*, *Rgs4*, *Mfap2*, *Marcksl1*), complement pathway activation (*Masp2*), and mitochondrial/cellular quality-control (*Fkbp8*), collectively pointing to structural and metabolic remodeling (55–59). Lipid metabolic and citrate-transport genes regulate membrane fatty-acid composition and intracellular citrate/Zn^2^^+^ levels, which are known to influence neuronal excitability, neurotransmission, and synaptic plasticity (60–63). Scaffolding and RGS proteins such as PATJ and RGS4 organize junctional and synaptic signaling complexes and tune GPCR/G-protein signaling at striatal synapses (64, 65), while MARCKS/MARCKSL1 family members regulate actin dynamics, spine structure, and neurite morphology (66, 67). Complement components including MASP2 contribute to developmental and insult-associated synaptic remodeling (68–70), and FKBP8 supports mitochondrial quality control as a mitophagy receptor and has recently been linked to mitochondria–ER contact site regulation and mitochondria complexity through PDZD8-FKBP8 tethering (71–73).

To identify the cellular pathways most affected by *Mfn2* deficiency, we first performed a mitochondrial gene set enrichment analysis (mito-focused GSEA). Mfn2^KO^ produced prominent depletion of mitochondrial pathways in both sexes, including those related to “mitochondrial organization,” “mitochondrial fusion and fission,” “respiratory processes,” and “regulation of membrane potential” (Fig. 4 *B* and *C* and *SI Appendix*, Table S2 in Dataset S1), indicating that mitochondrial structure and bioenergetics are major targets of *Mfn2* loss in D1-MSNs. Notably, in addition to these shared depletions, males also showed depletion of pathways associated with “oxidative phosphorylation,” “electron transport chain,” “pyruvate metabolic process,” and the “tricarboxylic acid cycle” (Fig. 4*D*), implying a greater disruption of energy metabolism in males compared to females.

To uncover additional pathways beyond these dominant mitochondrial effects, we next assessed enrichment among genes with nonmitochondrial function. Male Mfn2^KO^ mice showed a broad downregulation of ribosomal gene sets whith positive enrichment of synaptic and postsynaptic categories (Fig. 4*E* and *SI Appendix*, Table S3 in Dataset S1). Given the synaptic functional and structural alterations observed in Mfn2^KO^ males, we next asked whether the leading-edge (“core enrichment”) genes driving the enriched synaptic/postsynaptic GSEA terms could have roles in receptor trafficking and PSD remodeling machinery, and found that they indeed clustered into a coherent trafficking/PSD remodeling module (Fig. 4*F*). Specifically, the enriched synaptic signature included i) posttranslational regulatory mechanisms that control receptor stability and surface retention through ubiquitin- and lipid-dependent regulation, which together tune receptor trafficking and synaptic proteostasis (74–77); ii) clathrin-mediated endocytosis components that organize postsynaptic endocytic machinery for activity-dependent receptor internalization (78–80); iii) early endosome, cargo-sorting, and recycling pathways that control endosomal capture and recycling decisions for synaptic membrane cargos, including glutamate receptors (81–83); and iv―v) synaptic organizing programs that couple membrane trafficking to rearrangements in postsynaptic density architecture and cell adhesion (84–87). Together, these processes are established controllers of AMPA/NMDA receptor internalization and recycling and are tightly coupled to PSD turnover and spine structural plasticity (88–90). In contrast, no comparable enrichments were detected in females, indicating a sex-specific transcriptional response. The synapse-related enrichments in males align with the electrophysiological and morphological findings, supporting the idea of synaptic reorganization following Mfn2^KO^.

Collectively, DEG and GSEA analyses converge on a shared mitochondrial signature of *Mfn2* deficiency in both sexes, but males additionally show broader bioenergetic and ribosomal depletion with synaptic/postsynaptic enrichment, whereas females display a more circumscribed mitochondrial response biased toward structural and metabolic remodeling. Thus, for a comparable degree of *Mfn2* loss, male D1-MSNs appear to shift more toward stress- and damage-handling programs, whereas female D1-MSNs shift toward structural and metabolic reorganization, consistent with distinct sex-specific responses when adapting to *Mfn2* dysfunction.

### Mitochondrial Dysfunction in D1-MSNs Reconfigures Pathway Networks Linking Mitochondrial, Ribosomal, and Synaptic Programs.

We then examined potential interactions between the pathways that were significantly enriched in the two Mfn2^KO^ vs. WT GSEA runs, to deepen our understanding of how *Mfn2*-sensitive mitochondrial pathways relate to changes in synaptic and ribosomal pathways in male and female D1-MSNs. To this end, we first checked gene-to-gene correlations between pathways that were enriched in the mitochondria-focused GSEA vs. the GO-CC terms enriched in the second GSEA run. A clustered heatmap showing the fraction of significant gene–gene correlations over the total number of gene–gene pairs for each pathway pair revealed that the energy- and structure-related mitochondrial pathways showed the strongest links with ribosomal pathways, for both sexes (*SI Appendix*, Fig. S8 *A* and *C* and Table S4 in Dataset S1). Next, we determined the associations between all the enriched CC pathways in a similar manner and found the strongest links between ribosomal and synapse-related pathways for both sexes (*SI Appendix*, Fig. S8 *B* and *D* and Table S5 in Dataset S1). Together, these results support a model in which pathways responsive to *Mfn2* perturbations form a coordinated axis connecting changes in mitochondrial, ribosomal, and synaptic processes in D1-MSNs.

**Fig. 5.**
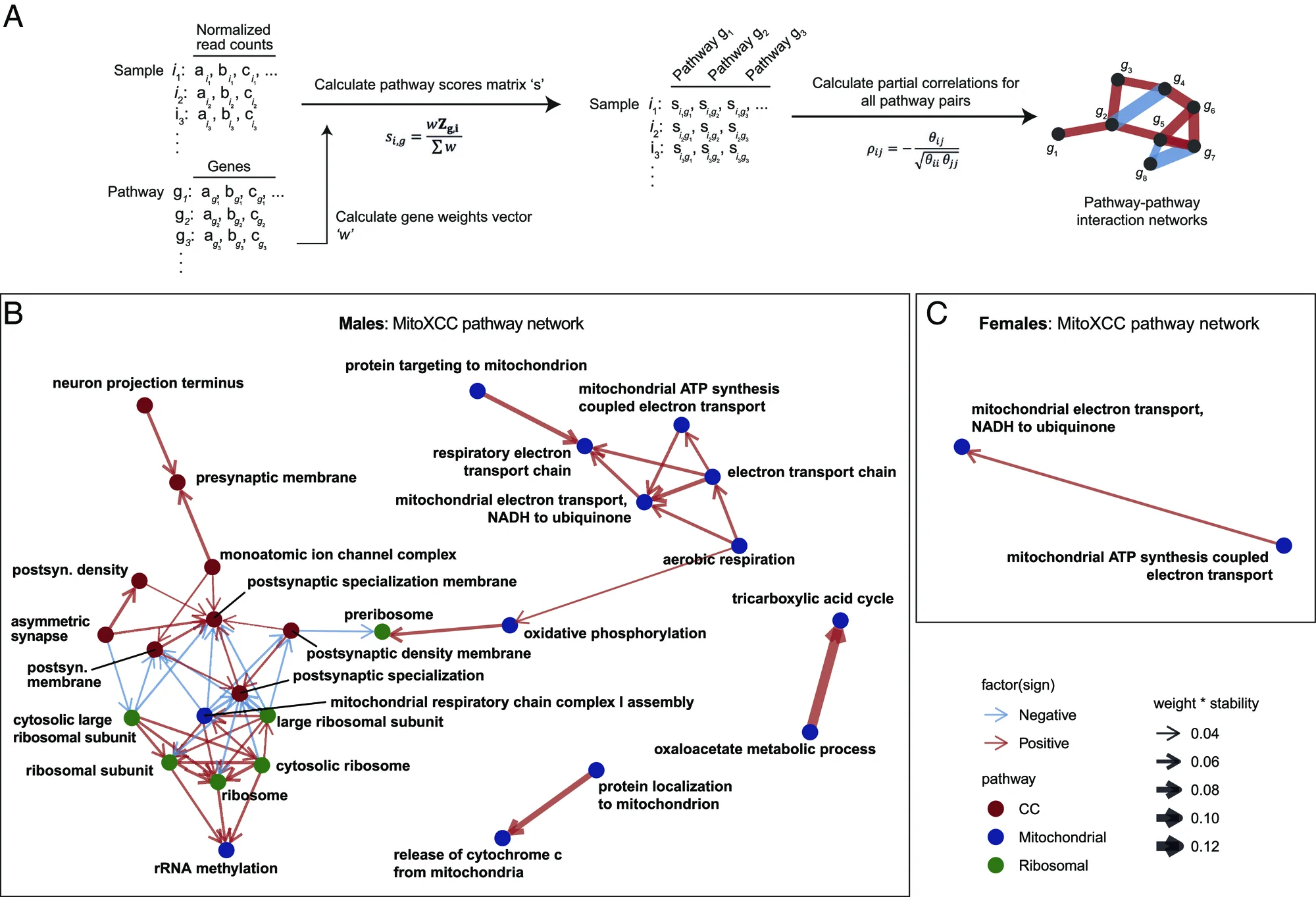
Investigation of sex-specific translatome changes reveals differences in pathway crosstalk in male and female mice upon Mfn2^KO^. (*A*) Bioinformatics pipeline for the pathway–pathway network inference. More information can be found in *Materials and Methods*. (*B*) Pathway network from the male dataset showing the most stable associations. Associations between pathways were defined by red or blue edges, signifying positive or negative regulation, respectively. The width of the edge reflects the product of weight of the association and the stability from the bootstrapping analysis. Nodes are colored by relevance as mitochondrial (dark blue), ribosomal (dark green), and rest of the GO-CC pathways (dark red). (*C*) Pathway network from the female dataset showing the most stable associations.

To further characterize these relationships, we employed a pathway-level network analysis to infer pathway–pathway association networks from gene expression data (Fig. 5*A*). We calculated pathway scores for the enriched GSEA pathways using weighted aggregates of normalized read counts and calculated sparse inverse covariance among pathway expression profiles. Finally, to identify robust pathway–pathway links, we ran a series of bootstrapping runs and retained only those links that appeared in more than 70% of the runs.

This analysis revealed a dense pathway network in males (Fig. 5*B* and *SI Appendix*, Table S6 in Dataset S1). Three main modules were evident: an energy metabolism cluster (*Upper Right*; mitochondrial electron transport, oxidative phosphorylation, TCA cycle, and related terms), a synaptic cluster (*Upper Left*; postsynaptic density, postsynaptic specialization, presynaptic membrane, asymmetric synapse), and a ribosomal cluster (*Lower Left*; ribosome, large ribosomal subunit, cytosolic ribosome, rRNA methylation). Notably, the mitochondrial pathway “mitochondrial respiratory chain complex I assembly” did not segregate with the core energy metabolism module but instead stay at the interface of the synaptic and ribosomal clusters, showing predominantly positive links with ribosomal pathways and negative links with postsynaptic terms. A similar pattern was observed for “preribosome,” which was positively linked to oxidative phosphorylation and negatively linked to ‘postsynaptic density membrane’, pointing to coordinated but opposing relationships between ribosomal and postsynaptic pathways.

In striking contrast, the corresponding network in females was extremely sparse (Fig. 5*C*), with a single stable edge connecting “mitochondrial ATP synthesis coupled electron transport” and “mitochondrial electron transport, NADH to ubiquinone” pathways, but no links involving synaptic or ribosomal pathways. These network-level analyses therefore indicate that, in the context of Mfn2^KO^, male D1-MSNs organize mitochondrial pathways within a broader interaction structure that links mitochondrial, ribosomal, and synaptic programs, whereas in females the stable associations remain largely confined to mitochondrial pathways, with no robust mitochondria-ribosome-synapse crosstalk detected. See *SI Appendix*, Table S7 for mito-focused GO-BP terms used for the mito-focused GSEA pipeline, and Dataset S2 for source data and statistical details for datasets presented in main figures and in *SI Appendix*.

## Discussion

Mitochondrial mechanisms within the nucleus accumbens have emerged as important determinants of motivated behavior, yet how *Mfn2* in D1-MSNs shapes effort-based motivation, and whether this differs by sex, has remained unclear. Here, we show how *Mfn2*-dependent mitochondrial integrity in ventral striatal D1-MSNs is required for appetitive effort allocation and active stress coping, and that males and females reach a shared motivational impairment through synaptic and molecular trajectories. Our data support a mitochondria-to-synapse model in which *Mfn2* loss compromises mitochondrial integrity and mitochondria–ER functional coupling, together with sex-specific deficits in synaptic structure and function, and consequent reductions in D1-MSN recruitment during motivated behavior, aligning with the observed motivational deficits. To probe the molecular underpinnings of this phenotype, we examined gene programs under translation in D1-MSNs using RiboTag and found that *Mfn2* loss reconfigures these programs in a sex-specific manner. In males, mitochondrial and ribosomal pathways are broadly depleted, while synaptic pathways are positively enriched, with the leading-edge genes including a prominent representation of receptor trafficking and PSD remodeling that is tightly coupled to glutamate receptor turnover and spine/PSD remodeling. Importantly, these enriched genes map onto critical synaptic control points (e.g., clathrin-mediated endocytosis, endosomal sorting and recycling, posttranslational regulation and proteostasis, synaptic organization, and receptor and scaffold cargo), providing a molecular bridge between *Mfn2*-dependent mitochondrial alterations and the synaptic phenotypes we observe. In females, mitochondrial pathway depletion predominates, without comparable changes in ribosomal and synaptic pathways, indicating a more circumscribed response. Thus, our findings support a model in which *Mfn2*-dependent mitochondrial integrity and/or its interactions with the ER set the capacity of this ventral striatal circuit to sustain motivated behavior, with sex-specific molecular adaptations converging in a common motivational deficit.

Notably, *Mfn2* deficiency produced region-specific consequences across striatal subdivisions. Whereas D1-MSNs in the dorsomedial striatum were largely spared from structural and functional changes, their NAc counterparts exhibited changes in excitatory input dynamics, dendritic complexity, and spine morphology. This regional specificity indicates that ventral striatal neurons are particularly dependent on *Mfn2*-mediated mitochondrial function, potentially reflecting differences in connectivity, firing patterns, and metabolic demands (91). Given that NAc D1-MSNs integrate limbic, cortical, and midbrain inputs encoding reward value, effort costs, and emotional salience (1, 22, 23), mitochondrial vulnerability in this region is well positioned to disrupt circuit computations required for effortful motivated behavior.

Consistent with this framework, Mfn2^KO^ mice showed impaired incentivized performance under high-effort conditions, while free feeding and low-effort performance remained intact, indicating a selective disruption of willingness to work for reward rather than alterations in basic incentive valuation, motor performance, or task acquisition. Increased passive stress coping responses, together with reduced cFos activation in accumbal D1-MSNs during task performance, further link *Mfn2*-dependent mitochondrial integrity in these neurons to the engagement of effortful motivation and active coping strategies. Together, the behavioral selectivity and reduced task-evoked recruitment argue that *Mfn2* loss does not simply “lower motivation” globally, but instead limits the ability of accumbal D1-MSNs to sustain the synaptic integration and circuit engagement required when effort costs are high.

A key aspect of our findings is that males and females exhibit similar neuronal recruitment deficits and behavioral consequences of *Mfn2* deficiency in D1-MSNs, yet reach these phenotypes through distinct circuit adaptations. In males, the overall pattern of synaptic and structural changes points to diminished excitatory and inhibitory synaptic drive and integrative capacity accompanied by structural retraction. In females, reduced excitatory input frequency and amplitude, increased inhibitory event frequency, preserved dendritic arborization, and a shift toward immature spines instead suggest altered postsynaptic efficacy and a reweighted excitation–inhibition (E–I) balance on a preserved dendritic structure. In both cases, these adaptations are expected to restrict D1-MSN participation in the ensemble activity supporting effortful behavior. This “degeneracy” in the effect of a common mitochondrial insult at the circuit level (i.e., where distinct synaptic and structural configurations converge on similarly reduced D1-MSN engagement) indicates that similar motivational phenotypes can arise from different underlying network states, an important consideration when inferring mechanisms or designing interventions targeting these circuits.

Cell-type-specific profiling of ribosome-associated mRNAs provides a mechanistic bridge linking *Mfn2* deficiency to these sex-divergent circuit phenotypes. Pathway-level analyses revealed a shared depletion of mitochondrial pathways related to mitochondrial dynamics, organization, membrane potential, and Ca^2+^ and ROS homeostasis in both sexes, consistent with *Mfn2*’s canonical roles at the outer mitochondrial membrane and ER–mitochondria contact sites (38, 42, 43). Importantly, only males showed additional depletion of core bioenergetic programs. Thus, for a comparable loss of *Mfn2*, male D1-MSNs exhibit a deeper energetic failure mode at the transcript level, whereas female D1-MSNs maintain expression of key respiratory and central carbon metabolism genes. Alongside this mitochondrial impact, ribosome-associated responses diverged sharply between sexes. In males, *Mfn2* deficiency led to a broad downregulation of ribosomal gene sets together with enrichment of synaptic/postsynaptic categories, consistent with a redistribution of biosynthetic investment under mitochondrial stress. This synaptic enrichment converged on a postsynaptic trafficking and PSD remodeling program encompassing endocytic mechanisms (80), endosomal sorting and cargo recycling for synaptic membrane receptors (83), posttranslational control of receptor stability and synaptic proteostasis (76, 77), and organizing programs that couple membrane trafficking to PSD architecture (87). Collectively, these processes regulate glutamate receptor surface cycling and are tightly linked to PSD turnover and spine structural plasticity (88–90, 92). Male-specific DEGs further indicated engagement of conserved stress-response pathways (protein quality control, endolysosomal function, apoptotic priming, DNA repair, and nutrient-sensitive O-GlcNAc signaling) (93–97), providing plausible routes by which mitochondrial disruption could reshape ribosomal and synaptic trafficking transcripts through energy- and redox-sensitive signaling that gates translation and proteostasis. In females, by contrast, *Mfn2*-sensitive DEGs clustered in lipid metabolism and citrate transport (55–58, 60–63), synaptic and junctional scaffolding (64–67), complement signaling (68–70), and mitochondrial and cellular quality control (59, 71–73), consistent with membrane- and signaling-centered remodeling rather than broad ribosomal suppression. While the positive enrichment of postsynapse-related pathways observed in the RiboTag dataset may seem counterintuitive considering our electrophysiological and immunofluorescence results in males, this increased abundance of ribosome-bound mRNAs encoding postsynaptic-related proteins may reflect a cellular attempt to compensate for the lower protein levels by boosting their translation.

Network inference further supports this sex divergence in pathway coupling. In males, mitochondrial, ribosomal, and synaptic pathways formed a dense interaction structure, and specific mitochondrial nodes (including complex I assembly) appeared at the interface between ribosomal and postsynaptic programs, suggesting that the degree and nature of bioenergetic pathways disruption are coupled to synaptic remodeling and translational control rather than representing parallel, independent consequences of *Mfn2* loss. In females, stable pathway associations were comparatively sparse and largely confined to mitochondrial terms, consistent with a more compartmentalized or heterogeneous downstream response.

Integrating these structural, physiological, and molecular findings, our data support a model in which *Mfn2* loss imposes a dendritic mitochondrial constraint that limits the capacity of NAc D1-MSNs to maintain and adapt synaptic integration during high-demand motivational states. In males, a deeper bioenergetic impact coincides with reduced ribosomal programs and engagement of postsynaptic trafficking and PSD remodeling signatures, consistent with a stress-linked translational recalibration (98), and aligns with the observed reductions in synaptic input dynamics and structural complexity. In females, relative sparing of core respiratory programs alongside changes in lipid metabolism, scaffolding, and mitochondrial quality control is consistent with maintained dendritic architecture but altered synaptic efficacy and E-I balance (60–72). Such sex divergence in mitochondrial and circuit responses is consistent with evidence that female mitochondria often exhibit greater respiratory capacity, more efficient Ca^2+^ buffering, and lower oxidative stress, in part driven by ovarian hormones (99–102), including estrogen support of respiratory chain function and antioxidant defenses (103–105), and sex differences in metabolic substrate utilization (101). Given the multiple roles of MFN2 in mitochondrial dynamics, mitochondria–ER communication, Ca^2+^ handling, and quality control, future work should define how these processes interact to shape D1-MSN synaptic remodeling and motivated behavior.

The present findings also connect mitochondrial regulation to contemporary models of mesolimbic control of motivation. Dopamine input to the NAc has long been implicated in the activational components of motivated behavior, including effort allocation, response vigor, and cost–benefit integration (2, 106, 107), while recent work indicates that the contributions of accumbal D1- and D2-MSNs to reward- and aversion-related motivational states may depend on their state-dependent firing patterns and ensemble recruitment rather than on fixed opponent roles (29–31). In this framework, *Mfn2*-dependent mitochondrial function emerges as a cell-intrinsic determinant of the synaptic input dynamics and task-evoked recruitment through which D1-MSNs translate motivational signals into sustained effortful action.

More broadly, our findings therefore have critical implications for understanding how mitochondrial disturbances within defined striatal cell types contribute to motivational phenotypes. They extend prior work linking accumbal MFN2 to affective and motivational phenotypes (32–36, 46, 108) by identifying D1-MSN MFN2 as a determinant of appetitive effort allocation and by delineating sex-related molecular programs through which mitochondrial disruption can reshape synaptic function and circuit recruitment. The coupling of motivational deficits with changes in ribosomal and synaptic remodeling pathways aligns with evidence that translational control and synaptic restructuring are recurrent features of depression-related brain states (22, 109), and supports the idea that mitochondrial dysfunction in defined ventral striatal cell types can contribute to individual differences in motivational capacity. Finally, the marked sex divergence cautions against inferring shared cellular mechanisms from shared behavioral outcomes and underscores the need to consider both sex and cell type when linking mitochondrial variation to motivation-related neuropsychiatric phenotypes.

## Materials and Methods

Details on the experimental procedures are contained in *SI Appendix*.

### Experimental Model.

Animal care and experimental procedures complied with Swiss Federal Guidelines for Animal Experimentation and were approved by the Cantonal Veterinary Office Committee for Animal Experimentation (Vaud, Switzerland).

All mice used in this study were on a C57BL/6 background. Unless otherwise noted, experiments were performed on B6.129(Cg)-Mfn2tm3Dcc/J (*Mfn2^lox/lox^*) mice (110). Both *Mfn2^lox/lox^ (Mfn2^KO^) and Mfn2^+/+^ (WT)* mice received stereotactic viral injections at 5 to 6 wk of age into the NAc to target D1-MSNs, as detailed in *SI Appendix*.

In a subset of experiments, male transgenic mice from the *Mfn2lox-Tg(D1-CreERT2)-R26-LSL-TdTom* line were used to induce conditional *Mfn2* downregulation in D1-MSNs. This line was generated by crossing *Mfn2^lox/lox^* mice with *D1-Cre^ERT^* (111) and *R26-LSL-tdTomato* (112) lines. Specificity of expression in D1 neurons has been confirmed for the NAc and medial striatum, with sparse expression observed in the dorsal and lateral striatum, as well as discrete regions in the cortex and ventral hippocampus (111). Both *Mfn2D1^ERT2+^* (referred to throughout the manuscript as Mfn2^cKO^) and *Mfn2D1^ERT2−^* (WT) mice were treated with tamoxifen between 4 and 7 wk of age. Tamoxifen (100 mg/kg at 5 µL/g) dissolved in sunflower oil was administered via daily injections for 5 consecutive days. Electrophysiological experiments were conducted 4 wk posttreatment.

Animals of the same genotype were grouped postweaning to minimize litter effects. Mice were housed in groups of 2 to 4 matched for age and body weight, in ventilated cages with ad libitum access to food and water. Mice were maintained on a 12-h light/dark cycle (lights on at 07:00 AM) in a temperature- and humidity-controlled environment (23 ± 1 °C, 50 ± 15% humidity). All behavioral testing occurred during the light phase, except for operant conditioning tasks. Behavioral and ex vivo experiments were conducted >4 wk after viral infection in mice aged between 10 and 20 wk.

### Statistics.

Fiji (v2.14) and QuPath (v0.5.0) were used for quantification of immunofluorescence and immunohistochemistry images. Statistical analyses were generally performed on GraphPad Prism (v10). Shapiro–Wilk tests were used for assessing normal distribution. Data following normal distribution were analyzed with Student’s unpaired *t* test or ANOVA accordingly to statistical needs. Data not following normal distribution were analyzed with the Mann–Whitney test or Kruskal–Wallis test. Grubbs’ test was used for identification of statistical outliers. Tukey’s test was used for post hoc analysis. For cumulative frequency analyses of synaptic events, the first 200 events recorded from each cell were included. Statistical comparisons between cumulative distributions were performed using the Kolmogorov–Smirnov test with a significance level of α = 0.001.

Bioinformatics analyses were performed in R (v4.3.3) using the DESeq2 package (v1.42.1). Differential expression analysis carried out with DESeq2 used an adjusted *P*-value cutoff of 0.1.

In the figures, data are presented as mean ± SEM, unless otherwise indicated in the figure legends. Datapoints in graphs represent single observations. Source data and statistical information are provided in Dataset S2.

## Supplementary Material

Appendix 01 (PDF)

Dataset S01 (XLSX)

Dataset S02 (XLSX)

## Data Availability

Source data for all figures, RiboTag sequencing data, full tables from the bioinformatics analyses, and the R scripts used for the analyses are publicly accessible in Figshare (https://doi.org/10.6084/m9.figshare.32252133) (113).
