# Supplementary material for: Mitofusin-2 in ventral striatal D1 neurons regulates effort-based motivation through sex-specific mitochondrial–synaptic reprogramming
Source: Proc Natl Acad Sci U S A. 2026 Jul 15;123(29):e2601657123. doi: 10.1073/pnas.2601657123 (PMC13389689; doi:10.1073/pnas.2601657123)
Supplement: Supplementary file 1 — Appendix 01 (PDF) [file pnas.2601657123.sapp.pdf]

PNAS

Supporting Information for

Mitofusin-2 in ventral striatal D1 neurons regulates effort-based motivation through sex-specific mitochondrial–synaptic reprogramming

Alessandro Chioino, Dogukan H. Ulgen, Olivia Zanoletti, Isabelle Guillot de Suduiraut, Ashley M. Maynard, Elisenda Sanz, Albert Quintana, Simone Astori, Carmen Sandi

Corresponding authors: Carmen Sandi, Simone Astori
Email: carmen.sandi@epfl.ch, simone.astori@epfl.ch

This PDF file includes:

Supporting text
Figures S1 to S8
Legends for Datasets S1 and S2
SI References

Other supporting materials for this manuscript include the following:

Dataset 1: Supplementary Tables
Dataset 2: Source Data and Stats

Supporting Materials and Methods

Genotyping

All mice were genotyped before weaning (from ear punches) by polymerase chain reaction (PCR) either in house or by outsource to an external company (Transnetyx, Tennessee, USA). The following primers were used for $Mfn2^{KO}$ and $Mfn2^{+/+}$ to detect the lox cassette: 5'-CCACGTTAGGCATTTGTG-3 (forward) and CGGCCGCGAATTCATAACTT (reverse). To detect control *Mfn2* gene, the following primers were used: 5'-TGTTTACTTTGGAAGTAGGCAGTCTC-3 (forward) and GGTATGAGCATGTGACCTGTTTCA (reverse). The $Mfn2^{D1ERT2+}$ and $Mfn2^{D1ERT2-}$ lines were additionally genotyped for: 5'-TCCTGGGCATTGCCTACAAC-3 (forward) and CTTCACTCTGATTCTGGCAATTTCG (reverse), to detect the Cre-Tax gene. To detect the conditional fluorescent reporter, TdTom: 5'-AGATCCACCAGGCCCTGAA-3 (forward1) and 5'-TTCCCTCGTGATCTGCAACTC-3 (forward2) and GTCTTGAACTCCACCAGGTAGTG (reverse1) and CTTTAAGCCTGCCCAGAAGACT (reverse2).

Brain surgery and viral vectors

$Mfn2^{KO}$ and WT mice were anesthetized with 4% isoflurane in O_2 (CombiVet animal gas anesthesia system; Rothacher Medical, Switzerland) for induction, followed by maintenance with 1.5–2% isoflurane. Analgesia (buprenorphine, 0.1 mg/kg) was administered subcutaneously before and after surgery. Surgical procedures commenced only after animals failed to show a paw withdrawal reflex upon pinching. Mice were positioned in a stereotaxic apparatus (David Kopf Instruments) on a 37.5°C heating mat. The head was shaved, and a 1:2 mixture of lidocaine (2%) and bupivacaine (0.5%) was applied along the incision site. Viscotear gel was used to protect the eyes. The surgical area was scrubbed with betadine, and a midline incision exposed the skull, which was cleared of the periosteum.

Bilateral craniotomies were performed using a surgical microdrill at the following coordinates relative to Bregma: A/P +1.4 mm; M/L ±0.7 mm; D/V -4.5 mm. To downregulate *Mfn2*, we injected AAV2-pDyn-Cre (4.3×10^{12} GC/ml; Bertarelli Foundation Gene Therapy Core Facility). For fluorescent reporter expression, we used AAV1-Flex-TdTom (2×10^{13} GC/ml; Addgene 28306), and for Ribotag experiments, AAV6-Dio-RPL22-3HA-YFP (7×10^{11} GC/ml; Bertarelli Foundation Gene Therapy Core Facility). A total of 300 nl of viral solution was infused at 0.2 µl/min using a stainless-steel needle (0.15 mm diameter) attached to a Hamilton syringe (Hamilton Bonanduz AG) driven by an infusion pump (Harvard Apparatus, ref 70-3333). The needle was left in place for 12 minutes post-infusion before being slowly withdrawn.

Post-surgical monitoring was conducted for 10 days, with Dafalgan (500 mg in 250 ml water, 200–300 mg/kg/day) provided in the drinking water for the first 4 days. Experiments were initiated 4 weeks post-surgery to allow for recovery and viral vector expression.

Behavioral paradigms

Free feeding

Animals were moved to a bigger home cage (30 x 20 x 42 cm), paired by two according to original home cage organization and separated by a grid to avoid social isolation. An automatized food dispenser (hereafter FED3 (1)) was made accessible on one side of the walls. In free-feeding modality, a 20 mg pellet (F0071, Bio-Serv) was made available to the animals every time the mice were collecting the previously delivered pellet. Animals were maintained on free feeding for 2 days, and total pellet intake was quantified each day in the morning shortly after lights on. Animals were otherwise left undisturbed.

Fixed ratio 1 (FR1)

In FR1 mode, the left poke was the "active" port, while the right poke was "inactive". The first correct nosepoke was designated as a poke performed in the active poke during hours of device activity. Thereafter, correct nosepokes also included pokes in the active port only after collecting the previously delivered reward and after a 5-s latency between two consecutive pokes. Upon each correct nosepoking, a pellet (5TUL, TestDiet®) was made available. Upon correct nosepoking, an auditory cue (4 kHz for 0.3 s) and a light cue (blue light from LEDs below food tray) were given. Animals were kept on FR1 for three or four consecutive days, and FED3 device was activated during dark phase (7:00 pm) for 12 or 6 h, depending on experimental needs. Animals were daily monitored, and the data transfer was performed daily shortly after lights on (7:00 am). Mice were moved to next training step when they showed they learned the task (i.e., stable performance across days).

Fixed ratio 3 (FR3)

After showing stable performance in FR1, mice were moved to FR3 mode in which three consecutive active pokes were required for the delivery of a pellet (5TUL, TestDiet®) in the food tray. FED3 device was active during dark cycle (7:00 pm – 7:00 am). Animals were moved to the next phase when displaying stable performance.

Progressive ratio (PR)

After FR1 and FR3 training, the animals were moved to a PR paradigm to test for motivated behavior. During the first exposure, mice were tested for a three hours session in which the operant requirement was integer (rounded to nearest) of the function:

response ratio= round((5 * exp((fed3.BlockPelletCount + 1) * 0.2)) - 5)

starting at one nose-poke. Two days later, animals were re-exposed to the same task but the device was kept on for the whole dark phase.

Forced Swim Test (FST)

The FST was employed to evaluate effort-based motivated behavior under aversive stimulation (i.e., inescapable stress). A transparent plastic cylinder, filled with 3.5 L of water at a controlled temperature of 23 ± 1°C, served as the FST apparatus. The animals were introduced and maintained in the apparatus for 6 min, after which they were removed from the water. Coping behavior was assessed by analyzing the %immobility during the 6 min of testing. Scoring was performed blind to the experimental conditions.

Elevated Plus Maze (EPM)

The EPM consisted of a central platform (5 × 5 cm) elevated 70 cm above the floor, from which two opposing open arms and two opposing closed arms (each 30 cm in length) extended. Illumination was maintained at 15–16 lx on the open arms and 5–7 lx in the closed arms. At the start of each trial, mice were gently placed on the central platform facing a closed arm and allowed to explore the maze freely for 5 min without disturbance. Following each trial, the maze arms were cleaned with 5% ethanol and dried thoroughly. Animal movement was recorded using a video camera mounted above the maze. Locomotor activity and anxiety-related behavior were analyzed using the EthoVision tracking system (EthoVision XT 11.0, Noldus Information Technology), which was used to calculate the total distance traveled and the percentage of time spent in the open and closed arms.

Open Field (OF) and Novel Object (NO) test

Mice were placed in the corner of a square arena (50 x 50 cm) and left to freely explore for 10 min (OF test phase). Afterwards, a new object was placed in the center of the arena for 5 min (NO test phase). Lighting was maintained at 8-10 lux on the center of the arena. After each trial, the arena was cleaned with 5% ethanol and dried. Video tracking of the animal's location was performed by a camera fixed above the arena. The total distance moved and the percentage of time spent in the center of the open field were measured with the Ethovision tracking system and taken as an indicator of anxiety (OF test phase) or reactivity upon novelty (NO test phase).

Light-dark box (LDB)

The light–dark box apparatus consisted of two compartments: a dark compartment measuring 20 x 60 cm and an illuminated (190 lux) compartment measuring 40 x 60 cm. Mice were placed in the dark compartment and given access to the lit compartment by removing a door. Behavior was recorded for 5 min, and the time spent in each compartment, as well as the number of transitions between compartments, was quantified as measures of anxiety-like behavior.

Ex-vivo experiments

Correlative-light electron microscopy

Following animal sacrifice, 80-μm-thick coronal brain sections were obtained using a vibratome. Sections were post-fixed in 1.5% potassium ferrocyanide and 2% osmium tetroxide, then stained with 1% thiocarbohydrazide and a second incubation in 2% osmium tetroxide. For confocal identification of D1-MSNs in the NAc, slices were maintained in PBS 1X and mounted onto SuperFrost Plus slides. Confocal imaging was performed on a Leica SP8 system. Initially, a low-resolution overview of the entire NAc was acquired using both brightfield and fluorescence (TdTom) channels with a 10x/0.30 NA air objective. Higher-resolution imaging of the same field was conducted using 20x/0.75 NA air and 40x/1.25 NA glycerol objectives. The same neurons were tracked across magnifications, and 1-μm z-stacks were acquired to capture both soma and dendritic processes. Anatomical landmarks, such as blood vessels, were used for consistent neuron identification across images.

Post-imaging, sections were stained overnight with 1% uranyl acetate, rinsed in distilled water at 50°C, and counterstained with lead aspartate (pH 5.0) at the same temperature. Tissues were dehydrated in graded ethanol series, embedded in Durcupan resin, and cured for 24 hours at 65°C between glass slides.

Serial block-face scanning electron microscopy (SBEM) was used to obtain ultrastructural data. The NAc-containing region was excised with a razor blade and mounted onto aluminum stubs using conductive glue. Blocks were trimmed with a glass knife and imaged using a Zeiss Merlin SEM equipped with a Gatan 3View microtome. Sections of 50 nm were sequentially removed from the block surface, and high-resolution images were acquired after each cut using an acceleration voltage of 1.7 kV, with 6.5-nm pixel size and 1-μs dwell time. Between 300 and 400 serial images were collected and aligned using FIJI (www.fiji.sc). Using the FIJI plugin TrackEM the dendrites were manually traced. Once completed, the dendrite labels were exported as .tiff files and saved. From the same TrackEM stack canvas, mitochondrial segmentation was performed using the trained general model EMPANADA-napari (https://empanada.readthedocs.io/en/latest/index.html). Labels of the detected mitochondria were saved as a .tiff file at the end of the analysis. The labels of both traced dendrites and mitochondria were imported in a new TrackEM file and overlapped on the initial stack image frame. Further morphological analysis was carried out in Blender (Blender.org), using the Neuromorph toolkit(2) to assess mitochondrial volume and length. Quantification was performed using stereological methods to estimate total mitochondria number. Mitochondrial density was calculated as the number of mitochondria per unit tissue area, while mitochondrial coverage was defined as the total area occupied by mitochondria relative to the tissue area. Dendritic spines were classified based on maximal head diameter, with thin spines defined as <0.3 μm and mushroom spines as >0.3 μm. Mitochondria-ER contact surface was

calculated using the Proximity Analysis tool with a threshold of (0.6 µm), below which the two organelles were considered in contact.

Electrophysiology

Coronal brain slices (250 µm thickness) were obtained from $Mfn2^{KO}$, $Mfn2^{cKO}$ and their respective WT littermates at age of 10-12 weeks using a vibrating tissue slicer (Campden Instruments). Mice were anesthetized on isoflurane, followed by decapitation. The brain was swiftly extracted and immersed in ice-cold modified artificial cerebrospinal fluid (ACSF), which was oxygenated with a mixture of 95% O_2 and 5% CO_2, containing (in mM): 105 sucrose, 65 NaCl, 25 $NaHCO_3$, 2.5 KCl, 1.25 NaH_2PO_4, 7 $MgCl_2$, 0.5 $CaCl_2$, 25 glucose, and 1.7 L(+)-ascorbic acid. Coronal brain slices containing the NAc were obtained and incubated in standard ACSF for 1 h at 35°C, the standard ACSF consisted of (in mM): 130 NaCl, 25 $NaHCO_3$, 2.5 KCl, 1.25 NaH_2PO_4, 1.2 $MgCl_2$, 2 $CaCl_2$, 18 glucose, 1.7 L(+)-ascorbic acid, and supplemented with 2 Na-pyruvate and 3 myo-inositol. In the recording chamber, slices were perfused with oxygenated standard ACSF either at room temperature. TdTom-positive MSNs in the NAc shell were patched in the whole-cell configuration using borosilicate pipettes (3-4 MΩ resistance). In $Mfn2^{KO}$ mice, recordings included also TdTom-positive neurons (D1-MSNs) in the dorsomedial striatum (DMS). Pipettes were filled with an intracellular solution containing (in mM): 120 CsGluconate, 10 HEPES, 0.2 EGTA, 10 phosphocreatine, 4 Mg-ATP, and 0.1% biocytin (290-300 mOsm, pH 7.2 to 7.3). Miniature excitatory postsynaptic currents (mEPSCs) were recorded at holding potential (-60 mV) in the presence of $GABA_AR$ blocker picrotoxin (0.1 mM) and the Na^+ channel blocker Tetrodotoxin (1 µM). To record spontaneous postsynaptic currents, 2.5 mM QX-314-Cl was added to the intracellular solution. For spontaneous excitatory postsynaptic currents (sEPSCs), MSNs were kept at -70 mV holding potential for 5 minutes. Neurons were then progressively moved towards positive potential and finally kept at +20 mV holding potential to record spontaneous inhibitory postsynaptic currents (sIPSCs) for 5 minutes.

At the end of the recording, the patch pipette was gently removed from the cell body to allow for the membrane to reseal. Slices were kept at 4% PFA overnight (O/N) and later moved to a 30% sucrose solution in PBS. Electrophysiological data were acquired using a Digidata1550A digitizer and amplified with a Multiclamp700B amplifier. Data were sampled at 20 kHz, filtered at 10 kHz, and recorded using Clampex10 software. Data analysis was conducted using Clampfit 10 software.

For mEPSCs, traces were low-pass filtered at 1 kHz, and event detection was performed using the MiniAnalysis Program (Synaptosoft Inc., Decatur, USA), with the detection threshold set at twice the root mean square (RMS) of the baseline noise. sEPCS and sIPSCs were detected from filtered traces (low-pass filtered at 1 kHz) and analyzed using Easy Electrophysiology v2.3 (Easy

Electrophysiology Ltd., UK) with the template matching method. A detection threshold of 5 pA was applied for event identification. All detected events were confirmed by visual inspection.

Brain tissue collection

For immunohistochemistry and RNAscope for WPRE, mice were anesthetized with a lethal dose of pentobarbital (150 mg/kg intraperitoneally; concentration <200 mg/ml, Esconarkon, Streuli Pharma) before transcardial perfusion with 0.9% saline, followed by fixation with 4% paraformaldehyde (PFA) in PBS (pH 7.5). Brains were rapidly extracted, stored overnight in 4% PFA, and rehydrated in 30% sucrose solution in PBS for at least 48 hours at 4°C. Tissues were snap-frozen in isopentane and stored at -80°C until further use. For cFos experiments, animals were sacrificed 90 minutes after the forced swim test (FST).

For RNA sequencing, animals were decapitated without prior anesthesia to avoid confounding effects on gene expression and neurotransmitter dynamics (3).Brains were immediately extracted, frozen in cold isopentane, and stored at -80°C until processing.

Immunofluorescence

Coronal brain sections (40–50 µm thick) were prepared using a sliding cryostat (3050S, Leica, Wetzlar, Germany) and stored in cryoprotectant medium at -20°C. For immunostaining, slices were rinsed in PBS and blocked for 1 hour at room temperature (RT) in a solution containing 3 -5% normal donkey serum (NDS, Jackson ImmunoResearch Laboratories, West Grove, PA, USA) and 0.3 - 0.5% Triton X-100 (Sigma-Aldrich). Sections were then incubated overnight at 4°C in antibody solution (PBS 1X, 0.3% Triton X-100, 3 - 5% NDS) containing rabbit anti-cFos (1:1000, Synaptic System 226008), rabbit anti-PSD95 (1:100, Cell Signaling 3450T), rabbit anti-GluN2b (1:50, Proteintech 21920-1-AP or rabbit anti-Shank2 (1:100, Cell Signaling 82529T). Anti-rabbit Alexa Fluor 647 (1:1000, Thermo Fisher Scientific ab2536183), anti-rabbit Alexa Fluor 488 (1:1000, Thermo Fisher Scientific A21206) were used as the secondary antibody and applied in antibody solution for 2 hours at RT. Nuclei were stained with 4,6-diamidino-2-phenylindole (DAPI, 1:10000 in PBS 1X) for 15 minutes at RT. After staining, sections were rinsed in PBS 1X and mounted on Superfrost glass slides (Thermo Fisher Scientific) using Fluoromount mounting medium (Southern Biotech, Birmingham, AL, USA).

For neuronal reconstruction from electrophysiology, slices were washed three times for 5 min in PBS 1X before being incubated O/N with streptavidin Alexa 488 (1:500, Thermo Fisher Scientific, S-11223) at 4°C in PBS solution supplemented with 0.05% Triton X-100. The day after, samples were washed in PBS 1X and mounted on SuperFrost Glass Slides.

For validation of injection sites, sections were stained only with DAPI to visualize nuclei.

RNAscope

Post-hoc in-situ hybridization (RNAscope Multiplex Fluorescent Reagent Kit v2, Advanced Cell Diagnostics, Inc.) was used to quantify *Mfn2* expression and assess viral targeting efficiency.

For quantifying *Mfn2* expression, 16 µm-thick brain sections were mounted on SuperFrost Plus slides, and RNAscope for fresh-frozen samples was performed per the manufacturer's protocol (Advanced Cell Diagnostics, Inc.). Briefly, sections were fixed in 4% pre-chilled PFA for 15 min at 4°C, followed by dehydration in 50%, 70%, and 100% ethanol for 5 min each at RT. Sections were air-dried for 5 min at RT, and hydrophobic barriers were drawn around them. After incubation with hydrogen peroxide for 10 min at RT, sections were washed with distilled water, treated with Protease IV for 30 min at RT, and rinsed in PBS. Probes to detect D1, D2, and *Mfn2* mRNAs (D1a, ref. 406491-C1; D2, ref. 406501-C2; *Mfn2*, ref. 581891-C3) were preheated and incubated for 2 h at 40°C. After washing, amplification steps using Multiplex FL v2 Amp1, Amp2, and Amp3 were performed sequentially at 40°C (30 min, 30 min, and 15 min, respectively). Signals were developed using Multiplex FL v2 HRP (15 min, 40°C) and Opal fluorophores (e.g., Opal 570 diluted 1:1500, Opal 690 diluted 1:750). Sections were counterstained with DAPI, mounted, and imaged. Signal amplification and development were repeated as needed for additional probes.

For quantifying viral targeting efficiency, 40 µm-thick perfused brain sections were processed similarly with modifications for fixed tissue. Briefly, sections were baked at 60°C for 30 min, dehydrated in graded ethanol, and incubated with hydrogen peroxide. Target retrieval solution was applied at 100°C, followed by incubation at 60°C for 10 min. Sections were then treated with Protease III for 30 min at RT. Probes for D1 and WPRE (Woodchuck Hepatitis Virus Posttranscriptional Regulatory Element, WPRE-O1-C2, no. 4502619) were applied and signal development was performed using TSA Vivid Fluorophore 570 (1:750 and TSA Vivid Fluorophore 650 (1:750), followed by DAPI counterstaining and mounting.

For electrophysiology-fixed samples, D1 and D2 mRNA expression was assessed qualitatively to identify cell types. Sections were processed similarly to the viral targeting efficiency protocol, except that target retrieval was reduced to 5 min before Protease III treatment (30 min, 40°C) to preserve both biocytin labeling and RNAscope signal quality.

Imaging and quantification

For cFos staining, brain slice images were acquired using a fluorescent whole-slide scanner (Olympus VS120) with a 20x/0.75 NA air objective. Three to four slices per brain area were collected along an anteroposterior gradient (Bregma +2.45 mm to -3.40 mm). The following channels were captured: DAPI, TdTom, and cFos. Quantification of cfos expression was performed using QuPath software(4). The NAc was identified according to the Allen Brain Atlas, and cell detection was performed based on the DAPI signal. Fixed intensity thresholds were applied for

cFos and TdTom to identify cFos-positive and TdTom-positive cells. For each animal, 3-4 brain slice images were analyzed, and the percentage of cFos-positive cells within TdTom-positive cells was calculated.

For PSD95, GluN2b and Shank2, brain slice images were acquired using a Leica SP8 confocal microscope with a 20X/0.75 NA air objective. Four images were analyzed using Fiji 1.54p. A TdTom -positive dendritic mask was generated by subtracting the DAPI mask from the TdTom signal, and mean synaptic marker fluorescence intensity was quantified within this mask.

For viral injection site validation, images were acquired with the VS120 (20x/0.75NA air objective) using DAPI and TdTom. Animals with viral spread outside the NAc were excluded from the analysis.

For neuronal morphological reconstructions post-electrophysiology, patched neurons were identified by biocytin expression and imaged with a confocal microscope (Leica SP8) using a 20x/0.75NA air objective. Morphology was captured in XY and Z axes (step size 0.3 μm). Sholl analysis quantified intersections radiating from the soma in 20-25 μm steps. For spine detection, 3–4 primary dendrites per neuron were imaged at their distal third using a 40x/1.25NA glycerol objective. Spine parameters were classified by morphology and head diameter (>0.3 μm, mushroom; <0.3 μm, thin) using an in-house script. Dendrites and spines were traced with the SNT plugin in Fiji.

For WPRE RNAscope, confocal images were acquired on an Leica SP8 confocal microscope with a 20X/0.75 NA air objective. Cell detection and mRNA quantification were performed using QuPath for cell segmentation and probe detection. Briefly, single nuclei were automatically detected based on the DAPI staining, and cell segmentation was performed with a cell expansion of 5 μm around the nucleus. After background fluorescence subtraction, probes for WPRE and Drd1 were detected as dots in each cell mask. Cells were classified as WPRE+ and Drd1+ if containing at least 5 and 3 dots of the corresponding probe, respectively, and WPRE- and Drd1- in the absence of any dot.

Primary culture generation and essays

Glass coverslips (Fisherbrand, 22-293-232P) were cleaned in water for 5 min, transferred to ethanol, flamed, and stored in culture dishes. Coverslips were autoclaved at 220°C for 2 hours, placed in 6-well plates, and incubated overnight with poly-D-lysine (Sigma-Aldrich, P6407; 1 mg/ml diluted 1:20 in PBS 1X) at 37°C. The next day, coverslips were washed three times with distilled water and rinsed three times with PBS 1X before incubation with laminin (Thermo Fisher Scientific, 23017015; 1.18 mg/ml diluted 1:96 in PBS 1X) at 37°C overnight. Laminin solution was removed the following day, and the coverslips were kept in medium until cell seeding.

Primary neuronal cultures were generated from P0/1 pups of Mfn2^{KO} and WT breeders. Pups of either sex were decapitated at room temperature (RT), and the skulls were opened using fine spring scissors and extra-fine forceps (Deutsche Biomedical, DBV1003 and DBF1005). Brains were placed in Hank's balanced salt solution (HBSS; Invitrogen Gibco, 14025-050) in a tissue culture dish (Techno Plastic Products AG, 93060). The striatum was dissected under a binocular stereo microscope (Motic, SMZ161) and transferred to a drop of digestion medium (8 mM L-cysteine, dissociation medium, 30% glucose, papain, pH 7.4; filtered through a 0.22 µm filter, Merck Millipore, SLGP033R). Tissue was collected in 5 ml of digestion medium and incubated at 34°C for 30 min.

Following digestion, samples were centrifuged at 2000 rpm for 2 min at RT. The supernatant was discarded, and the pellet was resuspended in 5 ml of protease inhibitor medium (dissociation medium, 30% glucose, trypsin inhibitor; filtered through a 0.22 µm filter) for 10 min at RT before another centrifugation step. The supernatant was discarded, and the pellet was resuspended in 4 ml of trituration medium (MEM, 30% glucose, 50 mM L-glutamine, horse serum; filtered as before). The pellet was mechanically triturated by pipetting 20 times with a Pyrex glass pipette. After sedimentation (~2 min), 2 ml of supernatant was transferred to a clean 15 ml tube. Trituration was repeated three times or until no visible debris remained, adding 3 ml of fresh trituration medium with each cycle. The final supernatant was centrifuged, and the pellet containing neurons and glia was resuspended in adhesion medium (MEM, 30% glucose, 50 mM L-glutamine, Penicillin/Streptomycin, horse serum).

Cells were counted using a Neubauer chamber (Blaubrand, 10.0442.01) with a 1:5 dilution in 0.4% trypan blue (Thermo Fisher Scientific, 15250061). Cell suspensions were plated at 400,000 cells/well in a 6-well plate and 30,000 cells/well in a 96-well plate using adhesion medium. Three to four hours after plating, the medium was replaced with growth medium (Neurobasal, B27, 50 mM L-glutamine, Penicillin/Streptomycin).

At DIV1, cell attachment and growth were verified. To reduce glial proliferation, Ara-C (Sigma-Aldrich, C6645) was added at DIV4 at a final concentration of 0.5 µl/ml from a 5X stock.

Viral infections were conducted at DIV2 to induce *Mfn2* knockdown (Mfn2^{KO}) and express fluorescent markers in D1-MSNs. Multiplicity of infection (MOI) was calculated for each viral vector, with AAV1-pDyn-Cre used at 10k MOI, a vector carrying fluorescent marker TdTomato at 30k MOI (AAV-Flex-TdTom, Addgene, 28306) and AAV6 MitoGCaMP5 at 30k MOI.

For 6-well plates, 2 ml of conditioned medium was removed from each well and set aside. Fresh growth medium containing the viral vectors was added to the remaining 2 ml in the plate. Plates were incubated overnight at 37°C with 5% CO_2. The following day, wells were washed twice by adding and removing 2 ml of equilibrated growth medium. After the final wash, the conditioned

medium was returned to each well to achieve a final volume of 4 ml. Experiments were conducted between DIV10-14.

Mitochondrial calcium was measured in cultures plated on glass coverslips in 6-well plates. Coverslips were transferred to a culture dish with equilibrated medium and maintained in the incubator until imaging. For recording, coverslips were placed in a sealed chamber, washed twice with Tyrode solution, and perfused with recording solution containing (in nM): 5 TTX, 1000 D-APV, and 200 DNQX. The chamber was mounted on a point-scanning microscope (Leica SP8 STED 3X) with temperature and CO_2 control. Cells were imaged using a 25×/0.95 NA water-immersed objective, with baseline fluorescence recorded for 5 min. A depolarizing solution (60 mM KCl) was applied, and imaging continued for 15 min. Fluorescence changes in mitochondrial calcium were quantified as ΔF/F over time and normalized to the reporter channel.

Mitochondrial membrane potential was assessed using tetramethylrhodamine methyl ester (TMRM, 150 nM), a fluorescent dye sequestered by active mitochondria. Coverslips were incubated with TMRM for 30 min at 37°C, washed twice with Tyrode solution, and perfused with recording solution. Baseline TMRM fluorescence was recorded for 5 min, followed by perfusion with a solution containing (in µM): 1 oligomycin and 1 FCCP. Imaging continued for 15 min. Changes in TMRM fluorescence were tracked over time and analyzed using Fiji (ImageJ, NIH). Mean intensity values were measured in regions of interest (ROIs) drawn on D1-expressing cells and expressed as ΔF/F. Photobleaching was corrected by normalizing the signal to a double exponential fit curve extrapolated from the baseline period.

Ribotag assay

The Ribotag pulldown was performed as previously described(5). Briefly, a bilateral injection of AAV-pDyn-Cre and AAV6-DIO-RPL22-3HA-IRES-YFP in the NAc followed by a sacrifice four weeks after ensured expression of the HA-tagged Rpl22 in a D1-MSN-specific manner. The NAc was punched from 200 µm-thickness slides, and the punches were homogenized in the Homogenization buffer (50 mM Tris–HCl pH 7.5, 100 mM KCl, 12 mM $MgCl_2$, 1% NP-40, 1 mM dithiothreitol, 200 U/ml RNasin (Promega, Madison, WI, USA, ref. N2111), 1 mg/ml heparin, 100 mg/ml cycloheximide, 1× protease inhibitor). After tissue clearance, the supernatant was incubated with anti-HA antibody (anti-HA.11 Epitope Tag Antibody, BioLegend, San Diego, CA, USA, ref. MMS-101R) bound to magnetic beads (Pierce Protein A/G magnetic beads, Thermo Fisher Scientific, ref. 88803) overnight at 4°C. The following day, four rounds of washes with a high-salt buffer (50 mM Tris–HCl pH 7.5, 150 mM KCl, 1% NP-40, 500 mg/ml cycloheximide, 12 mM $MgCl_2$) were carried out to remove non-specific interactions. After the last wash, beads were resuspended directly in RNA lysis buffer (from RNeasy Micro Kit, Qiagen, Netherlands; ref. 74004) and RNA isolation was carried out according to the manufacturer's instructions.

RNA quantification and integrity

mRNA was quantified by Qubit fluorometric quantification (Qubit 4 Fluorometer, Thermo Fisher Scientific, ref. 33238). Samples were in the range of 3–10 ng/µl, while undiluted inputs were up to 25 ng/µl. RNA integrity was assessed prior to library preparation by fluorescence-based electrophoresis (TapeStation 4200, Agilent Technologies, Santa Clara, CA, USA, ref. G2991A) using RNA High Sensitivity ScreenTape (Agilent, ref. 5067-5576) and RNA integrity number equivalent (RINe) was obtained. Samples with RINe above 7.5, were then used to generate cDNA libraries.

RNA library preparation and sequencing

RNA library preparation using NEBNext Ultra II Directional RNA Library Prep (New England BioLabs Inc., Ipswich, MA, USA, ref. E7760S) and sequencing using NovaSeq6000 (Illumina, Inc., San Diego, CA, USA, ref. 20012850) to obtain paired-end (100 bp) reads were outsourced to the Gene Expression Core Facility (GECF) at EPFL.

Reads were aligned to the mouse genome (grcm38) using HISAT2 with the '-rna-strandedness' parameter set to reverse reads(6). Read counts for each gene locus were obtained using the featureCounts function of the subread package (v2.0.2)(7). Low-abundance genes were removed before data normalization, keeping only genes with at least five reads in all samples. After normalizing the read counts by size factor, differential expression analysis was performed using the DESeq2 package in R (R Core Team, 2014)(8). P-values were corrected for multiple comparisons using the Benjamini–Hochberg method. Subsequent bioinformatics analyses were carried out using a custom-made script in R, using clusterProfiler package (v4.10.1)(9) for gene set enrichments. For extracting mitochondria-focused GO-BP terms, we checked the percentage of overlapping genes between all GO-BP terms and the mitochondrial function-related genes, as defined by MitoCarta 3.0. Pathways with at least 10 genes, with a higher than 50% overlap with mitochondrial function-related genes were picked for the subsequent analysis. This subset of pathways, with their genes, can be found in Dataset 1, Table S7.

Pathway-pathway network inference

To quantify functional interactions between biological pathways, we have developed a pathway–pathway network inference framework applicable to RNA-seq data. Briefly, normalized read counts were aggregated at the pathway level by computing weighted pathway scores per sample. For a given pathway g, the score for sample i was calculated as the weighted mean expression of all constituent genes:

$$s_{i,g} = \frac{w\mathbf{Z}_{\mathbf{g,i}}}{\sum w}$$

where $\mathbf{Z_{g,i}}$ denotes the scaled expression matrix of pathway genes across nuclei, and w is a gene-specific weight vector derived from variance stabilization. This procedure yields a pathway–by–sample matrix, reducing dimensionality.

To infer direct associations between pathways, we estimated a sparse inverse covariance matrix from the pathway score matrix using a graphical lasso approach (114, 115). For two pathways i and j, the corresponding partial correlation was defined as:

$$\rho_{ij} = -\frac{\theta_{ij}}{\sqrt{\theta_{ii}\,\theta_{jj}}}$$

where θ is the precision matrix. Non-zero edges in this graph represent conditional dependencies between pathways, controlling for all others, thereby distinguishing genuine co-regulation from indirect correlations.

To get an integrated pathway network including the stability of the links, we next bootstrapped the data and ran the analysis 100 times. We then only picked pathway links with ≥70% stability, and created an integrated network where the edge thicknesses indicate (weight * stability).

Figures

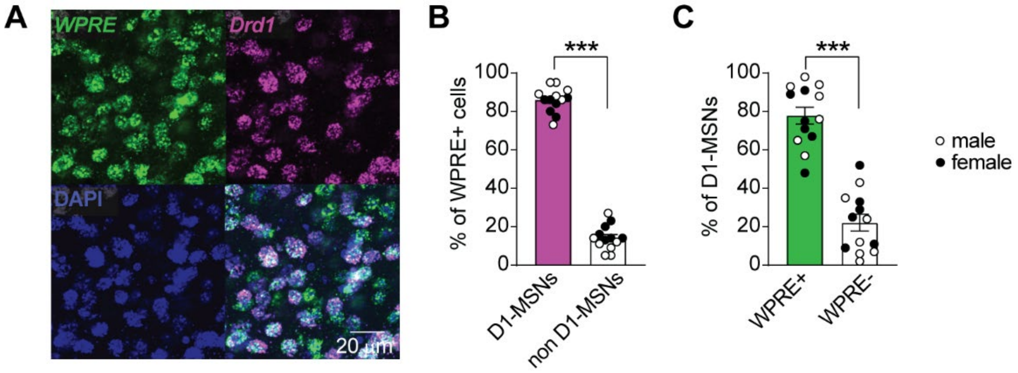

Fig. S1. Verification of viral approach for selective D1-MSN targeting (A) Confocal micrographs of RNAscope detecting WPRE and Drd1 mRNA in DAPI-stained NAc cells in Mfn2^{KO} mice. (B) Quantification of the % of WPRE+ (i.e., AAV-Dyn-Cre expressing cells), indicating specific targeting of D1-MSNs (i.e., cells expressing Drd1). (C) Quantification of the % of D1-MSNs cells expressing WPRE+ within infected regions, indicated that the large majority of D1-MSNs were targeted by the AAV.

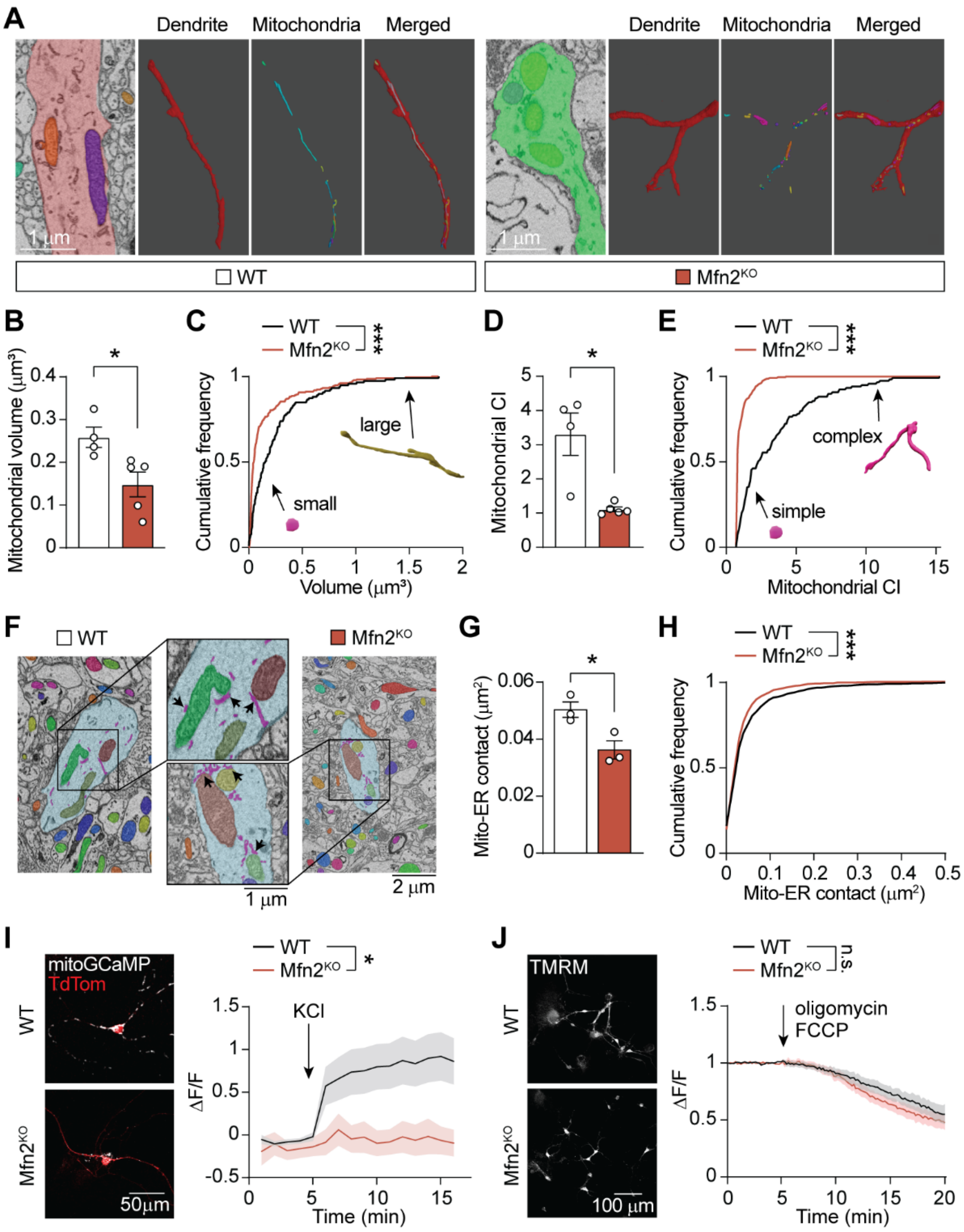

Fig. S2. **AAV-mediated Mfn2^{KO} in D1-MSNs disrupts core *Mfn2*-dependent mitochondrial and mitochondria-ER functions.** (A) Confocal and correlated light-electron microscopy (CLEM) approach used to identify TdTomato-labeled D1-MSN dendrites and reconstruct dendritic mitochondria. CLEM analyses in panels A-H were performed in male mice. (B) Quantification of mitochondrial volume showing significantly smaller mitochondria in Mfn2^{KO} D1-MSN dendrites (unpaired t test, $p = 0.02$). (C) Cumulative frequency distribution of mitochondrial volume, indicating a higher prevalence of smaller mitochondria in Mfn2^{KO} (Kolmogorov-Smirnov D = 0.33, $p < 0.0001$). (D) Quantification of mitochondrial complexity index (MCI), showing significantly reduced

mitochondrial complexity in $Mfn2^{KO}$ D1-MSN dendrites (unpaired *t* test, $p = 0.04$). (E) Cumulative frequency distribution of MCI highlighting reduced mitochondrial complexity in $Mfn2^{KO}$ neurons (Kolmogorov-Smirnov $D = 0.60$, $p < 0.0001$). (F) Representative CLEM images of dendritic segments from WT and $Mfn2^{KO}$ D1-MSNs, with traced mitochondria and endoplasmic reticulum (ER). Mitochondria-ER contacts are indicated by arrows in the insets. (G) Quantification of mitochondria-ER contact surface, indicating reduced contact surface in $Mfn2^{KO}$ D1-MSN dendrites compared with WT controls (unpaired *t* test, $p = 0.028$). (H) Cumulative frequency distribution of mitochondria-ER contact surface measurements, showing lower contact values in $Mfn2^{KO}$ dendrites (Kolmogorov-Smirnov $D = 0.082$, $p = 0.0007$). (I) Mitochondrial Ca^{2+} uptake in D1-neurons from primary striatal cultures generated from pups of both sexes. Representative mitoGCaMP responses following KCl-induced depolarization show reduced mitochondrial calcium uptake in $Mfn2^{KO}$ neurons compared with WT controls (repeated measures ANOVA for genotype effect, $F_{(1, 24)} = 5.25$, $p = 0.03$). (J) Mitochondrial membrane potential measured with TMRM in D1-neurons from primary striatal cultures generated from pups of both sexes, showing no significant differences between WT and $Mfn2^{KO}$ neurons (mixed-effects model for genotype effect, $F_{(1, 13)} = 0.63$, $p = 0.44$).

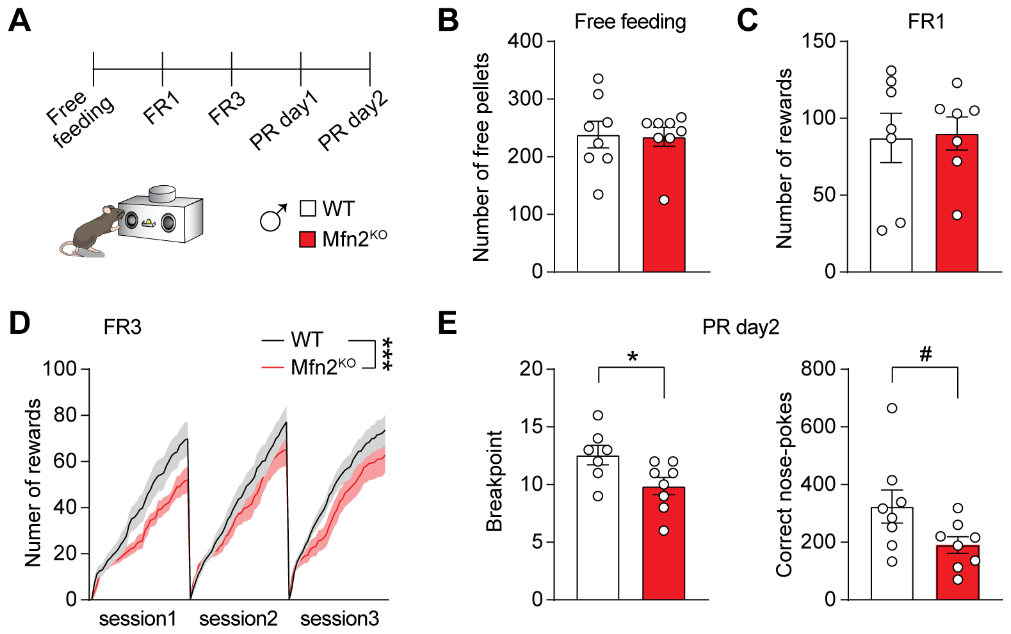

Fig. S3. Behavioral paradigm and control analyses for motivational tasks in male mice. (A) Experimental timeline of operant behavioral testing phases. (B) Number of free pellets consumed during free feeding showing no significant differences between groups (Mann-Whitney test, *p* = 0.96). (C) Number of rewards obtained during fixed ratio 1 (FR1) schedule, indicating no differences between WT and Mfn2^{KO} mice (unpaired *t* test, *p* = 0.88). (D) Performance over three sessions of fixed ratio 3 (FR3) schedule showing reduced reward acquisition in Mfn2^{KO} mice (repeated measures ANOVA for effect genotype $F_{(1, 1517)} = 150.7$, $p < 0.0001$). (E) Progressive ratio (PR) breakpoint (unpaired *t* test, *p* = 0.033) and correct nose-pokes (unpaired *t* test, *p* = 0.06), confirming significantly lower motivation in Mfn2^{KO} mice across sessions.

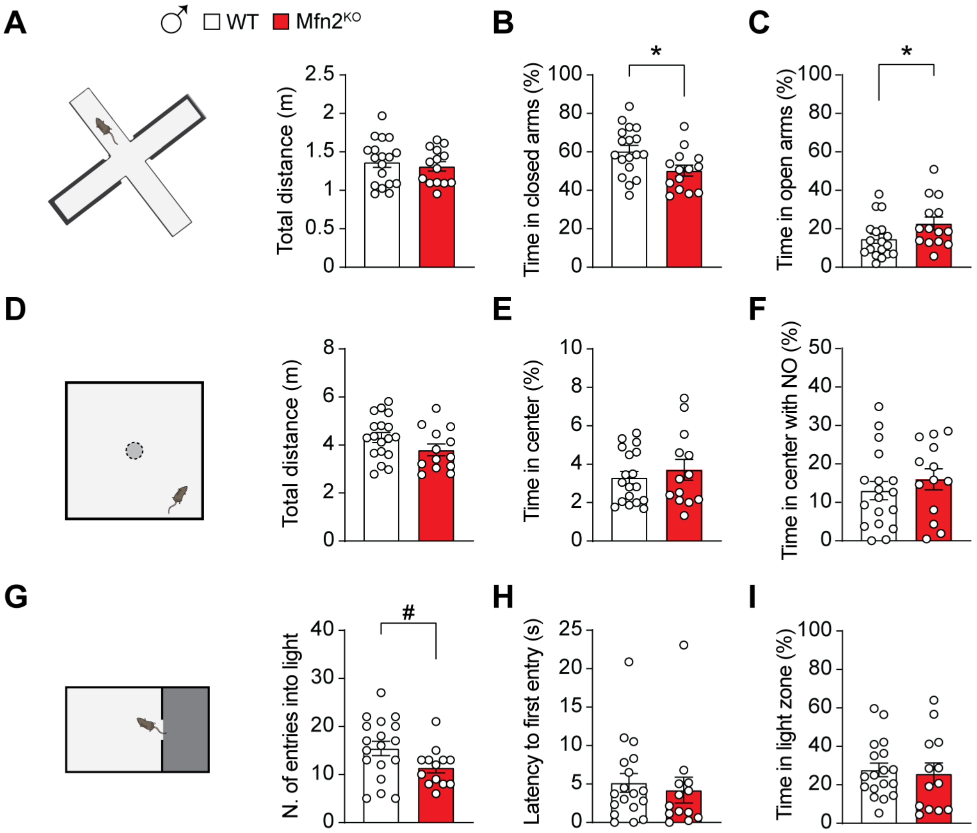

Fig. S4. Complementary behavioral paradigms in male mice. (A) Schematic representation of the elevated plus maze (EPM) apparatus and total distance moved, showing no difference between groups (unpaired *t* test, $p = 0.571$). (B) Mfn2^{KO} mice spent less time in the closed arms compared to WT mice (unpaired *t* test, $p = 0.021$). (C) Mfn2^{KO} mice spent more time in the open arms compared with WT mice (Mann-Whitney test, $p = 0.045$). (D) Schematic representation of the open field/novel object (OF/NO) apparatus and total distance traveled, showing no significant difference between groups (unpaired *t* test, $p = 0.119$). (E) Mfn2^{KO} mice spent a comparable amount of time in the center of the open field compared with WT mice (Mann-Whitney test, $p = 0.657$). (F) Mfn2^{KO} mice spent a similar amount of time exploring the novel object as WT mice (unpaired *t* test, $p = 0.423$). (G) Schematic representation of the light-dark box (LDB) apparatus and number of entries into the lit compartment, showing a tendency toward fewer entries for Mfn2^{KO} mice (unpaired *t* test, $p = 0.054$). (H-I) WT and Mfn2^{KO} mice showed comparable latency to enter the lit compartment (Mann-Whitney test, $p = 0.423$) and spent a comparable amount of time in the light zone (unpaired *t* test, $p = 0.76$).

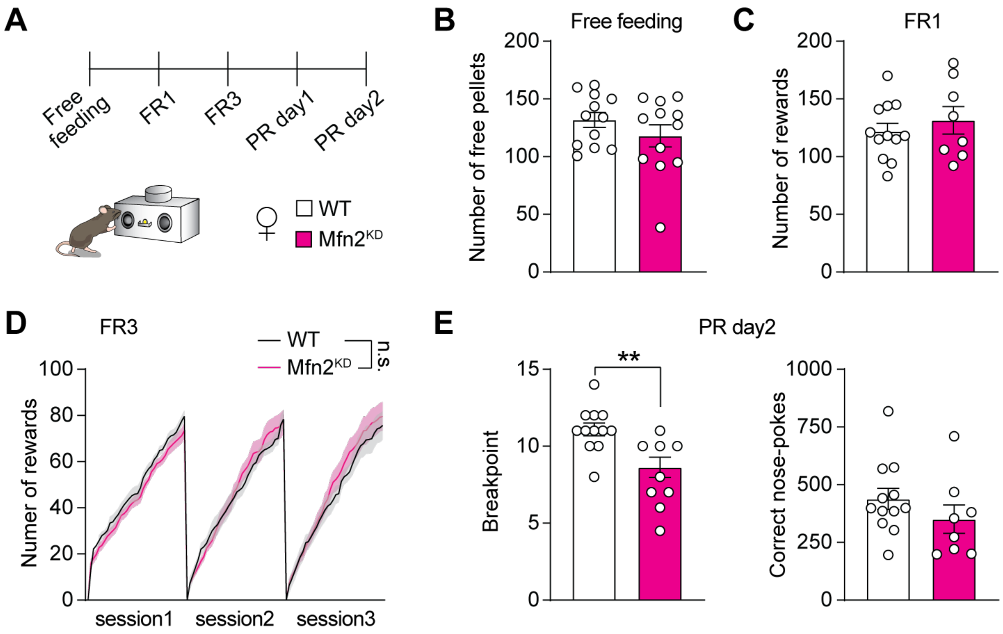

Fig. S5. Control behavioral analyses for motivational tasks in female mice. (A) Operant behavioral paradigm illustrating testing phases. (B) Number of free pellets consumed during free feeding showing no significant differences between groups (unpaired *t* test, $p = 0.25$). (C) Number of rewards obtained during fixed ratio 1 (FR1) schedule, indicating no differences between WT and Mfn2^{KO} mice (unpaired *t* test, $p = 0.5$). (D) Performance over three sessions of fixed ratio 3 (FR3) schedule showing comparable reward acquisition in WT and Mfn2^{KO} mice (repeated measures ANOVA for effect genotype $F_{(1, 1944)} = 0.33$, $p = 0.56$). (E) Progressive ratio (PR) breakpoint (unpaired *t* test, $p = 0.008$) and correct nose-pokes (unpaired *t* test, $p = 0.27$), confirming significantly lower motivation in Mfn2^{KO} mice across sessions. (F) Representative images of cFos immunoreactivity in female WT and Mfn2^{KO} mice after FST exposure.

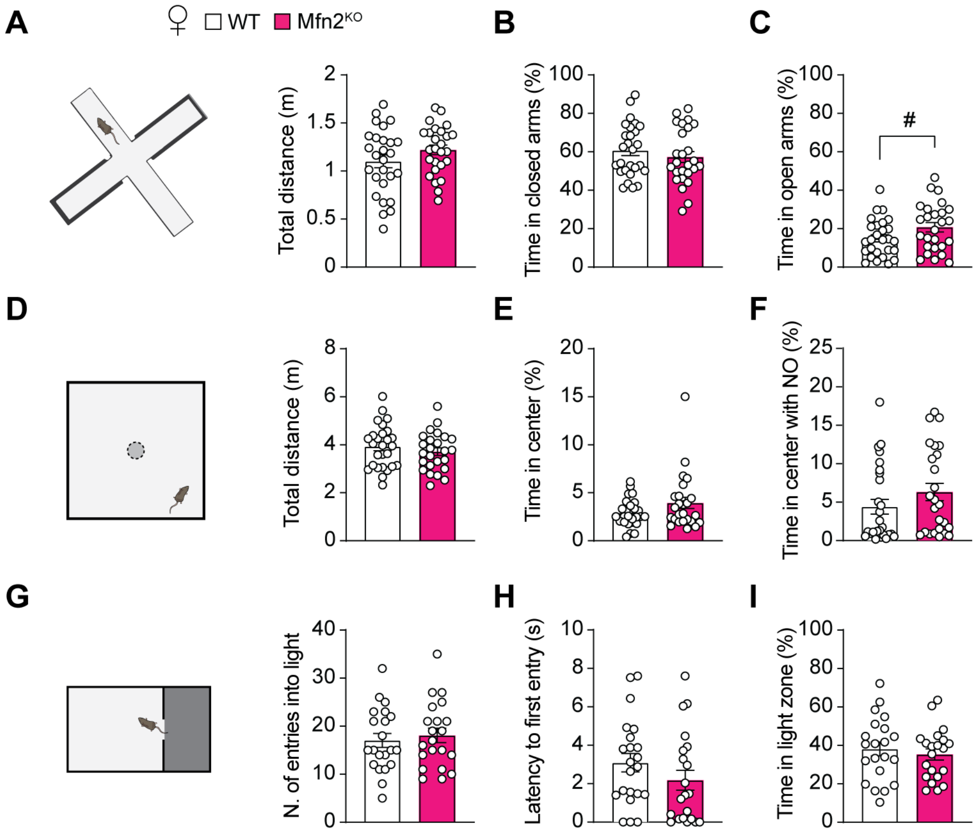

Fig. S6. Complementary behavioral paradigms in female mice. (A) Schematic representation of the elevated plus maze (EPM) apparatus and total distance moved, showing no difference between groups (unpaired *t* test, $p = 0.252$). (B-C) Mfn2^{KO} mice spent a comparable amount of time as WT mice in the closed arms (unpaired *t* test, $p = 0.39$) and showed a tendency toward increase time in the open arms (unpaired *t* test, $p = 0.066$). (D) Schematic representation of the open field/novel object (OF/NO) apparatus and total distance traveled, showing no significant difference between groups (unpaired *t* test, $p = 0.38$). (E) Mfn2^{KO} mice spent a comparable amount of time in the center of the open field compared with WT mice (Mann-Whitney test, $p = 0.32$). (F) Mfn2^{KO} mice spent a similar amount of time exploring the novel object as WT mice (Mann-Whitney test, $p = 0.116$). (G) Schematic representation of the light-dark box (LDB) apparatus and number of entries into the lit compartment, showing no difference between groups (unpaired *t* test, $p = 0.498$). (H-I) WT and Mfn2^{KO} mice showed comparable latency to enter the lit compartment (Mann-Whitney test, $p = 0.165$) and spent a comparable amount of time in the light zone (unpaired *t* test, $p = 0.71$).

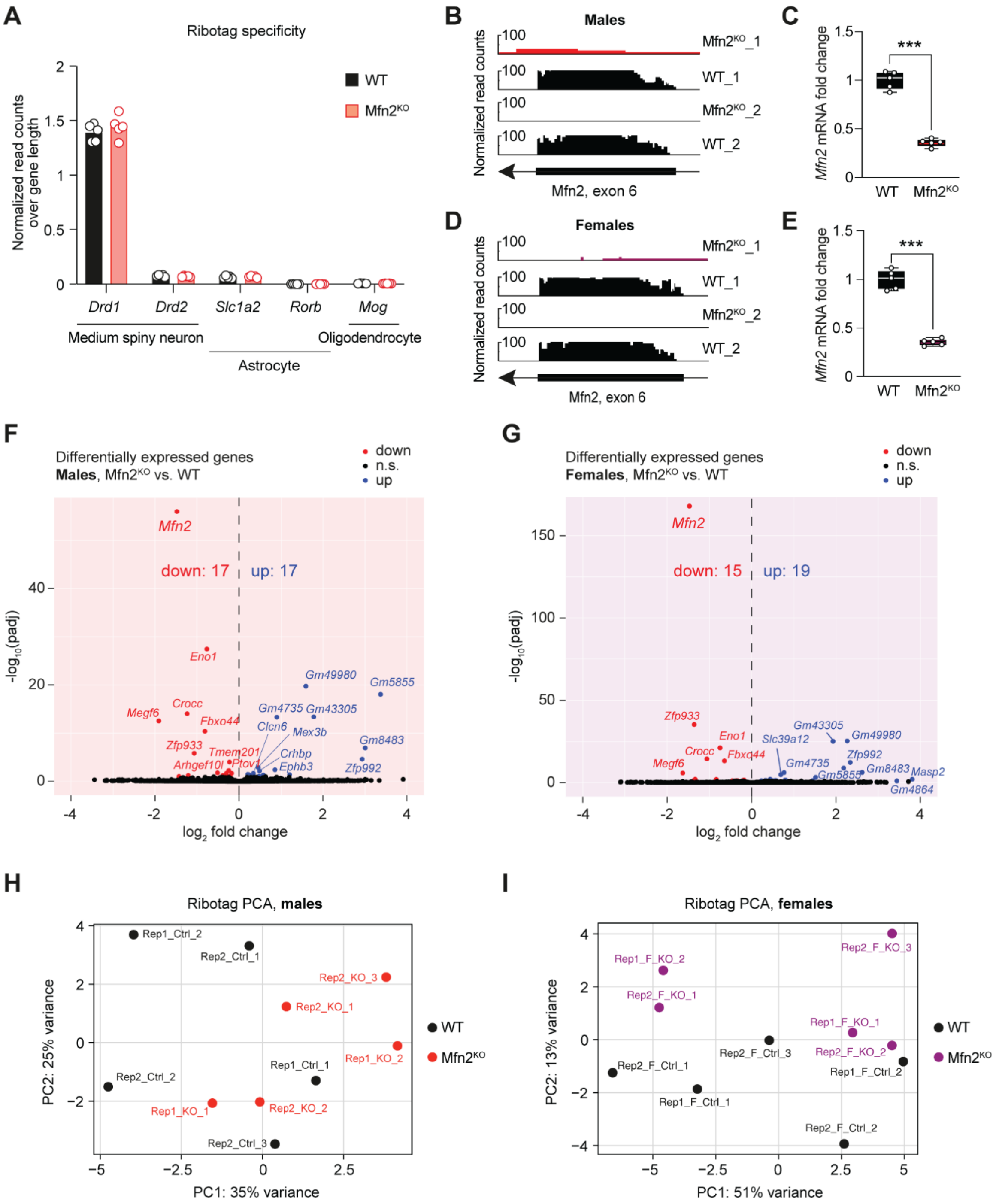

Fig. S7. Complementary data from the Ribotag sequencing in both sexes. (A) Normalized read counts of cell type-specific genes *Drd1*, *Drd2* (medium spiny neurons), *Slc1a2*, *Rorb* (astrocytes) and *Mog* (oligodendrocytes). The counts are also normalized over gene length. (B and D) Genome coverage of the *Mfn2* exon 6 region in four representative male and female Ribotag samples (2 WT, 2 Mfn2^{KO}). Exon 6 contained the lox/lox cassette which is excised in the Mfn2^{Dyn}

animals. (C and E) *Mfn2* reads in male and female Ribotag datasets. (F and G) Volcano plots of the differentially expressed genes in the Mfn2^{KO} vs. WT animals in males and females. A gene is indicated in red or blue, if significantly downregulated or upregulated in Mfn2^{KO}, respectively (padj < 0.1). (H and I) Principal component analysis (PCA) of the Ribotag samples in males and females.

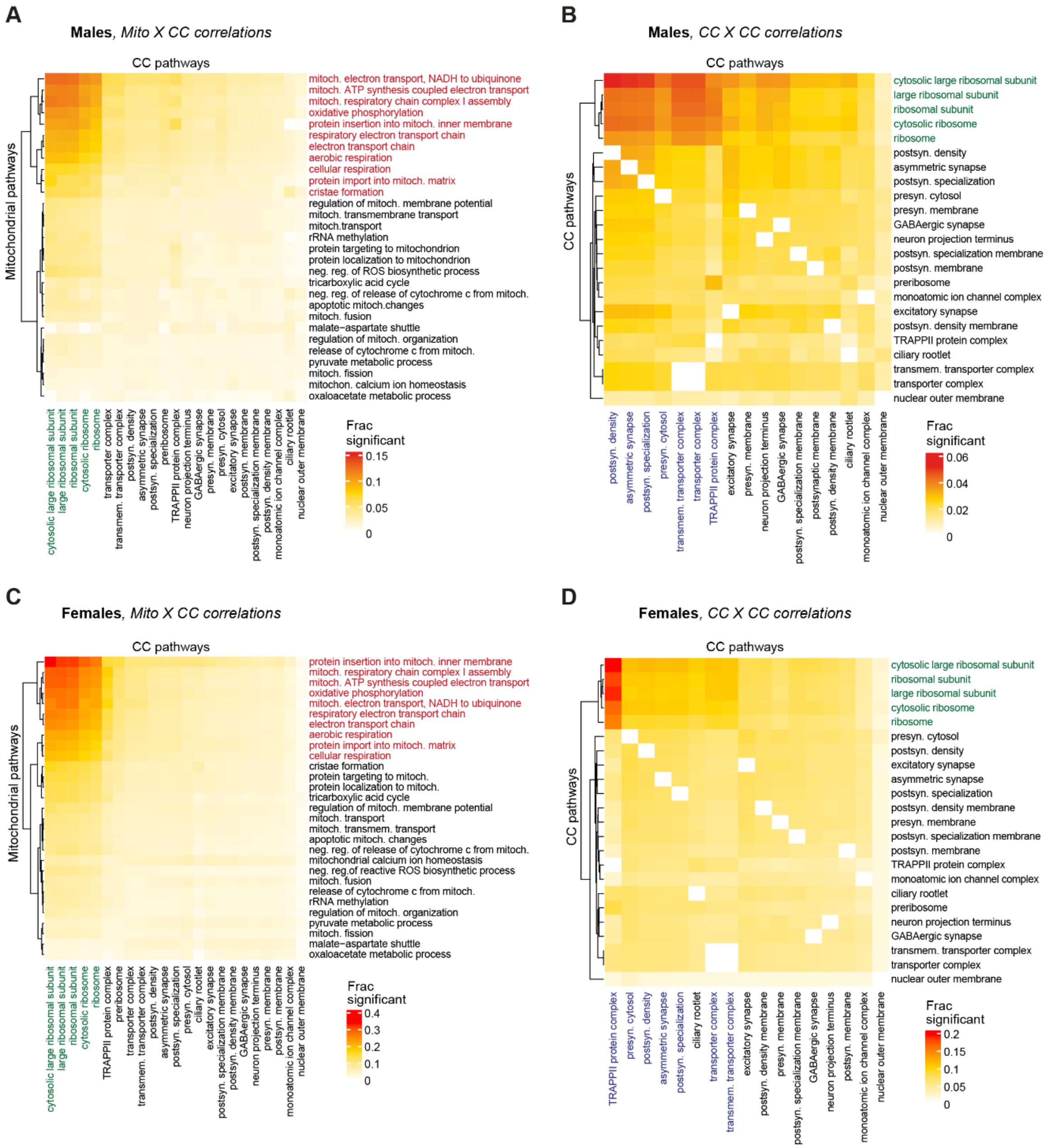

Fig. S8. Complementary data for the pathway investigations. (A and C) Heatmap of the fraction of significantly correlating gene pairs (p < 0.05) over the total number of possible correlations per pathway, for males and females. Enriched mitochondrial pathways in the mito-focused GSEA analysis (rows) were correlated to the enriched GO-CC pathways in males (columns). The same set of pathways were used for the female correlations, to provide a direct comparison. Rows and columns are hierarchically clustered to reveal the strongest correlations. (B and E) Same as A and C, but for GO-CC pathways only. Ribosomal pathways were removed from the columns, and same-same pathway correlations were replaced with a white box. Rows and columns are hierarchically clustered to reveal the strongest correlations.

Dataset 1 (separate file). Supplementary tables pertaining to the Ribotag RNA sequencing and bioionformatics analyses.

- Table S1: Ribotag differentially expressed genes in Mfn2^{KO} vs WT, for males and females.
- Table S2: Mito-focused GSEA results in males and females. This GSEA only includes GO Biological Process terms that are associated with mitochondrion or mitochondrial function (full list of pathways can be found in Table S7).
- Table S3: Non-mitochondrial GSEA results in males and females, after the removal of mitochondria-related genes from the dataset.
- Table S4: Gene-gene correlations between genes associated with mitochondrial or GO Cellular Component pathways that came out as significantly enriched in the previous analyses, for males and females. Fraction_Correlating_Genes show how many of the possible gene-gene pairs had significant correlations.
- Table S5: Gene-gene correlations between the significantly enriched GO Cellular Component pathways, for males and females. Fraction_Correlating_Genes show how many of the possible gene-gene pairs had significant correlations.
- Table S6: Integrated pathway-pathway networks between the mitochondrial and GO-CC pathways of interest. Edge weights are calculated using partial correlations (see Supporting Materials and Methods, Pathway-pathway network inference for more info) and stability of the interaction was calculated through bootstrapping. Combined score ('cs') is the geometric mean of ranked weights and stabilities.
- Table S7: Mito-focused GO-BP terms used for the mito-focused GSEA pipeline.

Dataset 2 (separate file). Source data and statistical details for datasets presented in main figures and in Supporting Information.

SI References

1. B. A. Matikainen-Ankney, *et al.*, An open-source device for measuring food intake and operant behavior in rodent home-cages. *Elife* **10** (2021).
2. A. Jorstad, J. Blanc, G. Knott, NeuroMorph: A Software Toolset for 3D Analysis of Neurite Morphology and Connectivity. *Front. Neuroanat.* **12** (2018).
3. I. Staib-Lasarzik, *et al.*, Anesthesia for Euthanasia Influences mRNA Expression in Healthy Mice and after Traumatic Brain Injury. *J. Neurotrauma* **31**, 1664–1671 (2014).
4. P. Bankhead, *et al.*, QuPath: Open source software for digital pathology image analysis. *Sci. Rep.* **7**, 16878 (2017).
5. D. H. Ulgen, *et al.*, Mitochondrial control of ciliary gene expression and structure in striatal neurons. *J. Physiol.* **0**, 1–21 (2025).
6. D. Kim, B. Langmead, S. L. Salzberg, Hisat2. *Nat. Methods* (2015). https://doi.org/10.1038/nmeth.3317.
7. Y. Liao, G. K. Smyth, W. Shi, The Subread aligner: Fast, accurate and scalable read mapping by seed-and-vote. *Nucleic Acids Res.* **41**, e108 (2013).
8. M. I. Love, W. Huber, S. Anders, DESeq2. *Genome Biol* (2014). https://doi.org/10.1186/s13059-014-0550-8.
9. T. Wu, *et al.*, clusterProfiler 4.0: A universal enrichment tool for interpreting omics data. *Innovation (Camb)* **2**, 100141 (2021).
